# Targeting the NSUN2–DHODH axis reverses ferroptosis resistance and oxaliplatin resistance in colorectal cancer

**DOI:** 10.3389/fphar.2026.1739981

**Published:** 2026-02-23

**Authors:** Junyi Zhang, Junxiao Shen, Tangye Zeng, Chang Gao, Biao Sheng, Junliang Li, Weiqi Zeng, Jieyi Shen, Chong Shen, Jiaojiao Wang, Jianwei Wang

**Affiliations:** 1 Department of Surgery, the Fourth Affiliated Hospital of School of Medicine, and International School of Medicine, International Institutes of Medicine, Zhejiang University, Yiwu, Zhejiang, China; 2 Department of Urology, the Fourth Affiliated Hospital of School of Medicine, and International School of Medicine, International Institutes of Medicine, Zhejiang University, Yiwu, Zhejiang, China; 3 Department of General Surgery, Sir Run Run Shaw Hospital, Zhejiang University School of Medicine, Hangzhou, Zhejiang, China; 4 Department of Oncology, the Fourth Affiliated Hospital of School of Medicine, and International School of Medicine, International Institutes of Medicine, Zhejiang University, Yiwu, Zhejiang, China; 5 Department of Radiation Oncology, Zhejiang Cancer Hospital, Hangzhou, Zhejiang, China; 6 Hangzhou Institute of Medicine (HIM), Chinese Academy of Sciences, Hangzhou, Zhejiang, China; 7 Zhejiang Key Laboratory of Particle Radiotherapy Equipment, Hangzhou, Zhejiang, China; 8 Department of Colorectal Surgery and Oncology, Key Laboratory of Cancer Prevention and Intervention, Ministry of Education, 2nd Affiliated Hospital, Zhejiang University School of Medicine, Hangzhou, Zhejiang, China

**Keywords:** 5-methylcytosine, colorectal cancer, DHODH, drug resistance, ferroptosis, NSun2, oxaliplatin

## Abstract

**Background:**

Oxaliplatin (OXA) is a standard chemotherapy for advanced colorectal cancer (CRC), yet acquired resistance frequently limits its efficacy. Ferroptosis, an iron-dependent form of cell death driven by lipid peroxidation, has emerged as a promising strategy to overcome chemoresistance. The RNA 5-methylcytosine (m5C) methyltransferase NSUN2 has been implicated in tumor progression, but its role in CRC chemoresistance remains unclear.

**Methods:**

We investigated the functional and mechanistic involvement of NSUN2 in CRC progression and OXA response, focusing on ferroptosis-related pathways. Integrative analyses of bulk, single-cell, and spatial transcriptomic datasets, together with multi-cohort clinical validation, were performed. Functional assays included colony formation, CCK-8 proliferation, migration, invasion, apoptosis, and xenograft experiments. Lipid ROS, malondialdehyde (MDA), and mitochondrial morphology were assessed to evaluate ferroptotic stress.

**Results:**

NSUN2 was upregulated in CRC and associated with poor prognosis. NSUN2 depletion suppressed CRC growth and enhanced sensitivity to OXA. Knockdown of NSUN2 increased lipid ROS accumulation, elevated MDA levels, and induced mitochondrial damage, consistent with enhanced ferroptosis. In vivo, NSUN2 depletion potentiated the antitumor activity of OXA in SW480 xenografts, and combining OXA with the ferroptosis inducer imidazole ketone erastin (IKE) further reduced tumor burden compared with OXA alone, accompanied by increased tumor MDA levels. Mechanistically, NSUN2 stabilized dihydroorotate dehydrogenase (DHODH) mRNA via m5C modification, thereby increasing DHODH expression. Elevated DHODH suppressed ferroptosis independently of GPX4, whereas NSUN2 depletion disrupted this axis, promoting lipid peroxidation and ferroptosis sensitivity. DHODH restoration rescued ferroptosis and reversed the enhanced drug sensitivity induced by NSUN2 knockdown.

**Conclusion:**

These findings identify an NSUN2–DHODH epitranscriptomic axis that promotes CRC progression and OXA resistance by limiting ferroptosis, supporting NSUN2-targeting and ferroptosis-inducing strategies to improve chemotherapy response.

## 1 Introduction

Colorectal cancer (CRC) is the third most commonly diagnosed malignancy and the second leading cause of cancer-related deaths worldwide, accounting for approximately 1 million deaths annually Bray et al. (2024). Chemotherapy remains a cornerstone in the treatment of CRC (Morris et al., 2023; Wang F. et al., 2025). As a third-generation platinum-based chemotherapeutic agent, oxaliplatin is a key component of first-line treatment regimens for advanced or recurrent CRC, as well as adjuvant therapy following radical surgery (Morris et al., 2023; Lu et al., 2025; Lieu et al., 2019). However, despite initial responsiveness, approximately 50% of patients with stage II or III CRC eventually develop resistance to oxaliplatin-based adjuvant chemotherapy, posing a major challenge to clinical management (Siegel et al., 2023; Väyrynen et al., 2020; Goldberg et al., 2004). Therefore, identifying key molecular drivers and underlying mechanisms of oxaliplatin resistance is critical for developing novel therapeutic strategies to address the challenges in CRC treatment.

Ferroptosis is a distinct form of programmed cell death that is iron-dependent and mechanistically different from apoptosis, necrosis, and autophagy (Tang et al., 2021). It is characterized by the accumulation of lipid peroxides on cell membranes and is governed by a dynamic balance between oxidative and antioxidant systems (Feng et al., 2023; Kuang et al., 2020). The initiation of ferroptosis relies on alterations in iron homeostasis, lipid metabolism, and antioxidant defense, and it is regulated by several pathways, including SLC7A11/glutathione (GSH)/glutathione peroxidase 4 (GPX4), FSP1/Coenzyme Q10 (CoQ10), Dihydroorotate dehydrogenase (DHODH)/Coenzyme Q10H2 (CoQH2), and GCH1/Tetrahydrobiopterin (BH4) axes (Li et al., 2022; Bersuker et al., 2019; Mao et al., 2021; Kraft et al., 2020). Cancer cells can evade ferroptosis, contributing to disease progression and therapeutic resistance, whereas induction of ferroptosis has emerged as a promising strategy to overcome chemoresistance (Jiang et al., 2021; Lei et al., 2024; Shen et al., 2024; Deng et al., 2025). For instance, CDK1 confers oxaliplatin resistance in CRC by promoting ubiquitin-mediated degradation of ACSL4, thereby suppressing lipid peroxidation and ferroptosis (Zeng et al., 2023). In addition, colorectal cancer stem cells (CCSCs) play a central role in mediating chemoresistance (Das et al., 2020). Histone lactylation enhances the expression of glutamate-cysteine ligase (GCLC), which inhibits ferroptosis signaling and lipid peroxidation, thereby promoting oxaliplatin resistance in CCSCs (Deng et al., 2025).

DHODH is a mitochondrial flavin-dependent iron-containing enzyme localized to the outer surface of the inner mitochondrial membrane (Barnes et al., 1993). As a rate-limiting enzyme in the *de novo* pyrimidine biosynthesis pathway, DHODH catalyzes the oxidation of dihydroorotate (DHO) to orotate (OA), concurrently reducing coenzyme Q (CoQ) to ubiquinol (CoQH2), thereby suppressing mitochondrial lipid peroxidation and ferroptosis (Sørensen and Dandanell, 2002; Löffler et al., 1997; Zhou et al., 2021). These findings highlight the essential role of DHODH in the mitochondrial defense against ferroptosis and suggest that DHODH inhibition may represent a promising therapeutic approach to trigger ferroptosis in cancer cells (Cao et al., 2025).

RNA 5-methylcytosine (m5C) modification is an important post-transcriptional regulatory mechanism (Wyatt, 1950; Trixl and Lusser, 2019; Desrosiers et al., 1974; Dubin and Taylor, 1975). NOP2/Sun RNA methyltransferase family member 2 (NSUN2), a key m5C methyltransferase, is overexpressed in various malignancies, including bladder, prostate, kidney, cervical, esophageal, gastric, liver, thyroid, and breast cancers (Yang et al., 2017; Li et al., 2024; Wang J. et al., 2025; Mao et al., 2025; Xue et al., 2022). Emerging evidence suggests that NSUN2 regulates m5C modification on specific mRNAs, thereby influencing cell proliferation, differentiation, and migration, and promoting tumor progression in an m5C-dependent manner (Lu et al., 2024; Chen et al., 2025). In this study, we found that NSUN2 was significantly upregulated in CRC tissues compared to adjacent normal tissues. NSUN2 depletion inhibited CRC cell proliferation *in vitro* and *in vivo*. Notably, silencing NSUN2 sensitized CRC cells to oxaliplatin, suggesting a role in chemoresistance. Further bioinformatic and mechanistic analyses revealed that NSUN2 is a key regulator of ferroptosis resistance. Moreover, our *in vivo* data indicate that NSUN2 depletion potentiates oxaliplatin-mediated tumor suppression, and that oxaliplatin combined with imidazole ketone erastin further reduces tumor burden, supporting the premise that augmenting lipid peroxidation–linked stress may enhance chemotherapy response. Mechanistically, NSUN2 enhances DHODH expression via m5C-mediated mRNA stabilization, which suppresses lipid peroxidation and ferroptosis; overexpression of DHODH partially rescued ferroptosis induced by NSUN2 depletion. Together, our findings suggest that NSUN2 is a promising therapeutic target for overcoming ferroptosis and oxaliplatin resistance in CRC.

## 2 Materials and methods

### 2.1 Bulk transcriptomics integration and processing

14 publicly available CRC bulk transcriptomic cohorts comprising tumor and adjacent normal tissues with clinical and prognostic information were integrated (Supplementary Table S1). Cohorts with sample size <50, non-original data, or incomplete clinical annotation were excluded. The Cancer Genome Atlas Colon Adenocarcinoma (TCGA-COAD) (n = 491) was obtained from the GDC portal (https://gdc.cancer.gov/), and 13 GEO datasets (GSE103479, GSE14333, GSE17536, GSE18105, GSE21510, GSE25010, GSE28702, GSE29621, GSE29638, GSE39084, GSE71187, GSE72970, and GSE87211) were downloaded from GEO (https://www.ncbi.nlm.nih.gov/geo/) (Cancer Genome Atlas Network, 2012; Allen et al., 2018; Jorissen et al., 2009; Smith et al., 2010; Matsuyama et al., 2010; Tsukamoto et al., 2011; Ågesen et al., 2011; Tsuji et al., 2012; Ågesen et al., 2012; Bruun et al., 2018; Kirzin et al., 2014; An et al., 2015; Cherradi et al., 2019; Hu et al., 2018). Among these, a prognostic meta-validation cohort comprising seven datasets with three types of clinical outcome records (total n = 1,550) was established. The remaining datasets were applied to analyses of clinical subgroup differences, pathway enrichment, and drug sensitivity. For cross-cohort comparison of target gene expression, raw data were normalized using log2(count + 1) transformation.

### 2.2 Single-cell transcriptomics integration

To elucidate the tumor biological characteristics of CRC, this study integrated 54 tumor and normal colon samples from four datasets (GSE166555, GSE299427, GSE232525, GSE200997) (Uhlitz et al., 2021; Wong et al., 2025; Yang et al., 2023; Khaliq et al., 2022) (Supplementary Table S2). Cell annotation was performed using a multi-tiered prioritization approach: (i) adopting original labels when available (GSE166555, GSE299427); (ii) manual annotation based on reference cell markers supplied within the original datasets (GSE232525, Supplementary Table S3); and (iii) confirming epithelial and stromal populations using established CRC epithelial markers (GSE200997, Supplementary Table S4). Data normalization, variable gene selection, and principal component analysis (PCA) were performed using the “Seurat” R package (Hao et al., 2024). Neighborhood graphs were constructed from PCA results, followed by Louvain clustering (resolution 0.3–0.5) and UMAP visualization. Batch effects were corrected using the “Harmony” algorithm.

### 2.3 Spatial transcriptomics processing

The spatial dataset GSE225857 was obtained from GEO (Wang et al., 2023) (Supplementary Table S2). Data normalization was performed using the “Sctransform” method to reduce technical artifacts while preserving biological heterogeneity. Downstream analyses involved clustering procedures analogous to those used in single-cell omics. Finally, spot annotation was guided by pathologist-provided histological labels, and spatial gene expression patterns were visualized using “SpatialFeaturePlo”.

### 2.4 Identification of differentially expressed genes (DEGs)

Differential expression analysis was performed using the “limma” algorithm. DEGs were defined as those with adjusted p < 0.05 and |log2FC| > 0.5. Ranked gene lists from each dataset were integrated using the ‘RobustRankAggreg’ (RRA) algorithm to identify genes consistently differentially expressed across cohorts (Kolde et al., 2012).

### 2.5 Survival analysis and meta-analysis

The “survival” package was utilized to calculate the hazard ratio (HR), 95% confidence interval (CI), log-rank p-value, and Kaplan–Meier (KM) curves. Meta-analysis was conducted using the “MIME” package, applying fixed- or random-effects models with the DerSimonian–Laird estimator to account for heterogeneity (Liu et al., 2024). Effect sizes were pooled via inverse-variance weighting. Heterogeneity was quantified using Q, I^2^, and H statistics, and publication bias was assessed via Egger’s test.

### 2.6 Functional enrichment and pathway activity scoring

Gene Set Enrichment Analysis (GSEA), Gene Set Variation Analysis (GSVA), and single-sample GSEA (ssGSEA) were performed using “clusterProfiler” and “gsva” R packages (Yu et al., 2012). For bulk data, non-significant genes (p > 0.05) were removed prior to GSEA. GSVA and ssGSEA were used to evaluate Hallmark gene sets and pathway activity at the single-cell level.

### 2.7 Multi-omics analysis of drug sensitivity

Drug sensitivity analysis was inferred using the “oncoPredict” package (bulk transcriptomics) and the “beyondcell” package (spatial transcriptomics) (Fustero-Torre et al., 2021). Specifically, for bulk samples, drug screening data were obtained from the Cancer Therapeutics Response Portal (CTRP) and the Genomics of Drug Sensitivity in Cancer (GDSC). Linear regression models were built for each drug and applied to CRC expression matrices to predict the half-maximal inhibitory concentration (IC50) values. For spatial transcriptomic samples, the “beyondcell” algorithm was applied to identify cells or spots with distinct drug response profiles. A Beyondcell Score (BCS) ranging from 0 to 1 was calculated for each drug–cell/spot pair, with higher scores indicating greater sensitivity to the specific drug.

Additionally, the Subclass Mapping (SubMap) algorithm was used to compare patient expression profiles with reference datasets of immune therapy responses (Hoshida et al., 2007). Samples were stratified based on either target gene expression patterns or immune response phenotypes to assess commonalities between subgroups. This analysis supported its utility in predicting differential responses to immunotherapy across distinct subgroups.

### 2.8 Statistical analysis and visualization

All statistical analyses and data visualization were performed using R (version 4.3.2) or Python (version 3.13). Continuous variable correlations were evaluated using Pearson’s correlation. Group comparisons were performed using the Wilcoxon rank-sum test. Classifier performance was assessed by Receiver Operating Characteristic (ROC) analysis, with area under the curve (AUC) indicating discriminatory ability. All statistical tests were two-sided, with p < 0.05 considered significant.

### 2.9 Cell lines and reagents

Human CRC cell lines SW480 and HCT116 and human embryonic kidney HEK293T cells were obtained from the American Type Culture Collection (ATCC, United States). Cells were cultured in Dulbecco’s modified Eagle’s medium (DMEM; Gibco, Thermo Fisher Scientific, Carlsbad, CA) supplemented with 10% fetal bovine serum (FBS; Gibco) at 37 °C in a humidified atmosphere with 5% CO_2_. Cells were routinely passaged at 70%–80% confluence using trypsin-EDTA and were used for experiments during the logarithmic growth phase. Unless otherwise specified, cells were seeded 12–24 h before treatment/transfection to achieve ∼50–70% confluence at the start of experiments. Oxaliplatin (L-OHP), erastin, Ferrostatin-1 (Fer-1), RSL3, Brequinar (BQR), and Actinomycin D were purchased from MedChemExpress (MCE, NJ, United States). Stock solutions were prepared according to the manufacturers’ instructions and stored aliquoted at −20 °C or −80 °C to avoid repeated freeze–thaw cycles. The working concentrations and treatment durations for each assay are provided in the corresponding figure legends.

### 2.10 Clinical samples

Primary CRC tissues and paired adjacent normal tissues were collected from patients at the Fourth Affiliated Hospital, Zhejiang University School of Medicine (2024–2025). Diagnosis was confirmed independently by two pathologists. Written informed consent was obtained from all participants. The study was approved by the Institutional Ethics Committee (K2025210) and conducted in accordance with the Declaration of Helsinki.

### 2.11 Plasmids, lentivirus production, and transduction

Lentiviral vectors encoding two independent NSUN2-targeting shRNAs (shNSUN2-1 and shNSUN2-2) or a scrambled control (shCtrl) were purchased from GeneChem (Shanghai, China). The target sequences were: shNSUN2-1: 5′-GAT​CGG​AGC​GAT​GCC​TTA​GGA​TAT​TAC​TCG​AGT​AAT​ATC​CTA​AGG​CAT​CGC​TCT​TTT​TT-3′; shNSUN2-2: 5′-GAT​CGT​GCA​GTG​TCC​CAT​CGT​CTT​ATC​TCG​AGA​TAA​GAC​GAT​GGG​ACA​CTG​CAT​TTT​TT-3′. Lentiviruses were generated in HEK293T cells using psPAX2 and pMD2.G packaging plasmids, and viral supernatants were harvested 48 h post-transfection. Target cells were infected at a multiplicity of infection (MOI) of 10–20, followed by puromycin selection (2 μg/mL, 7 days). NSUN2 siRNAs and negative control siRNA were obtained from GenePharma (Shanghai, China), with the following sequences: siNSUN2-1: forward 5′-GAG​CGA​UGC​CUU​AGG​AUA​UUA​tt-3′ and reverse 5′-UAA​UAU​CCU​AAG​GCA​UCG​CUC​tt-3′; siNSUN2-2: forward 5′-UGA​GAA​GAU​GAA​GGU​UAU​UAA​tt-3′ and reverse 5′-UUA​AUA​ACC​UUC​AUC​UUC​UCA​tt-3′. Constructs for NSUN2 wild-type (WT), enzymatic-dead mutants (C271A, C321A), and DHODH-WT were purchased from GenePharma. Transfections were performed using jetPRIME (Polyplus-transfection, Illkirch, France) according to the manufacturer’s protocol. Unless otherwise specified, cells were harvested for downstream analyses 24–72 h after transfection depending on the assay.

### 2.12 Wound healing and cell invasion assay

For wound healing assays, cells were seeded in 12-well plates and cultured to a confluent monolayer. A linear wound was created using a sterile 200-µL pipette tip, followed by gentle PBS washing to remove detached cells. The medium was then replaced with serum-free DMEM to minimize the confounding effect of cell proliferation, and images were acquired at 0 h and 24 h or 30 h at pre-marked positions using an inverted microscope. Wound closure was quantified by measuring the wound area in ImageJ, and migration was expressed as the percentage of wound closure relative to baseline (0 h).

For transwell invasion assays, Matrigel-coated transwell inserts (8-µm pore size; Corning, United States) were used. Briefly, 2 × 10^4^ cells were seeded into the upper chamber in serum-free medium, whereas complete medium containing serum was added to the lower chamber as a chemoattractant. After 24 h, non-invaded cells were removed from the upper surface with a cotton swab. Invaded cells on the lower surface were fixed, stained with crystal violet, imaged, and counted under a microscope. Quantification was performed using representative fields per insert, and values were averaged across biological replicates.

### 2.13 Cell viability assay

Viability was determined using the Cell Counting Kit-8 (CCK-8, Beyotime, China). Cells were seeded into 96-well plates and allowed to attach overnight. After the indicated treatments, CCK-8 reagent was added to each well and incubated for 2 h at 37 °C. Absorbance was measured at 450 nm using a microplate reader. Background values from medium-only wells were subtracted, and viability was normalized to the corresponding control group.

### 2.14 Colony formation assay

Cells were seeded into 6-well plates at low density and cultured for 10–14 days until visible colonies formed, with medium refreshed every 2–3 days. Colonies were fixed and stained with crystal violet. Colonies containing more than 50 cells were counted using ImageJ, and colony numbers were normalized to the control group.

### 2.15 Flow cytometry for apoptosis and lipid peroxidation

Apoptosis and lipid peroxidation were quantified by flow cytometry. For apoptosis analysis, cells were stained using an Annexin V-FITC/PI apoptosis detection kit (Meilunbio, China) and analyzed on a CytoFLEX S flow cytometer (Beckman Coulter, United States). Apoptotic cells were defined as Annexin V–positive populations. Compensation and gating were set using unstained and single-stained controls and applied uniformly across samples. For lipid peroxidation, lipid reactive oxygen species (lipid ROS) were assessed using BODIPY 581/591 C11 (Beyotime, China). Cells were incubated with the dye as recommended, washed, and analyzed.

### 2.16 Malondialdehyde (MDA) measurement

Malondialdehyde (MDA) levels were determined using a Lipid Peroxidation MDA Assay Kit (Beyotime, China). Cells or frozen tumor tissues were lysed or homogenized in Beyotime Western and IP cell lysis buffer (Beyotime, China), followed by centrifugation to collect supernatants for MDA determination. Total protein concentrations were measured using a BCA assay (Beyotime, China) for normalization, and MDA levels were expressed as nmol/mg protein.

### 2.17 Transmission electron microscopy (TEM)

TEM was used to examine mitochondrial morphology in cells treated with DMSO or erastin. After ethanol dehydration and resin embedding, samples were sectioned, stained and observed.

### 2.18 Quantitative real-time PCR (qRT–PCR)

RNA was extracted using TRIzol (Invitrogen, United States) and reverse-transcribed with HiScript III (Vazyme, China). qRT-PCR was performed with SYBR Green (Vazyme) on a CFX-96 amplifier (Bio-Rad, United States). GAPDH was used as an internal control, and expression was calculated by the 2^−ΔΔCT^ method. NSUN2 primers were 5′-ACA​GCC​ACT​GAG​TTG​GTA​TCC-3′ (sense) and 5′-TTA​TGA​TGA​GGC​CGC​ACG​TT-3′ (antisense). DHODH primers were 5′-CCA​CGG​GAG​ATG​AGC​GTT​TC-3′ (sense) and 5′-CAG​GGA​GGT​GAA​GCG​AAC​A-3′ (antisense). GPX4 primers were 5′-GAG​CCA​GGG​AGT​AAC​GAA​GAG-3′ (sense) and 5′-TGG​TGA​AGT​TCC​ACT​TGA​TGG-3′ (antisense). GAPDH was used as an internal control for normalization of the results. GAPDH primers were 5′-AGG​TCG​GTG​TGA​ACG​GAT​TTG-3′ (sense) and 5′-GTA​GAC​CAT​GTA​GTT​GAG​GTC​A-3′ (antisense).

### 2.19 Western blotting

Proteins were extracted using RIPA lysis buffer supplemented with PMSF (Beyotime, China), separated by SDS-PAGE, and transferred onto PVDF membranes (Millipore, United States). Membranes were blocked with 5% non-fat milk dissolved in TBST at room temperature for 1 h, and then incubated with primary antibodies against NSUN2 (20854-1-AP, Proteintech), GPX4 (ab125066, Abcam), DHODH (ab24689, Abcam), and β-actin (66009-1-Ig, Proteintech) at 4 °C for 12 h. After washing with TBST, membranes were incubated with HRP-conjugated secondary antibodies (EpiZyme) at room temperature for 1 h. Bands were visualized using an ECL detection system (Bio-Rad) and quantified by integrated density using ImageJ, with β-actin used as a loading control.

### 2.20 RNA dot blot assay

For dot blot, total RNA was denatured at 95 °C for 10 min in a metal bath and immediately chilled on ice. Denatured RNA was then spotted onto nylon membranes, followed by UV crosslinking at 254 nm for 1 h. Membranes were blocked with 5% non-fat milk in TBST at room temperature for 1 h, and incubated with an anti-m5C antibody (68301-1-Ig, Proteintech) at 4 °C for 12 h. After washing with TBST, membranes were incubated with HRP-conjugated secondary antibodies at room temperature for 1 h. Methylene blue staining was used to verify equal RNA loading, and signals were detected using an ECL detection system (Bio-Rad).

### 2.21 RNA stability assay

For RNA stability, cells were treated with 5 μg/mL actinomycin D (MCE) and harvested at 0–3 h for qRT-PCR analysis of DHODH mRNA decay.

### 2.22 Animal experiments

All animal procedures were approved by the Animal Experimental Ethics Committee of Zhejiang University (ZJU20250668). Four-week-old male BALB/c nude mice (GemPharmatech, Jiangsu, China) were maintained under specific-pathogen-free (SPF) conditions. For the subcutaneous xenograft model, SW480 cells (5 × 10^6^) in 0.1 mL PBS were injected subcutaneously into the right flank. Tumors were measured every 2 days, and volume was calculated as L × S^2^/2. For growth validation, mice were randomized into shCtrl and shNSUN2 groups (n = 5 per group); mice were euthanized at a predefined endpoint (tumor volume of 2,000 mm^3^), and tumors were excised, photographed, and weighed. For treatment evaluation, mice were randomly assigned to five groups (n = 5 per group) before inoculation and injected with shCtrl- or shNSUN2-transduced SW480 cells according to group assignment. Oxaliplatin (OXA) was dissolved in sterile 5% glucose solution, and imidazole ketone erastin (IKE; MCE, NJ, United States) was prepared in 10% DMSO vehicle. Treatment was initiated for each mouse when its tumor volume reached approximately 100 mm^3^, with the first dosing day defined as treatment day 0. The groups were as follows: vehicle control (shCtrl tumors; i.p. vehicle), oxaliplatin (shCtrl tumors; OXA 5 mg/kg i.p. every 3 days), NSUN2 knockdown (shNSUN2 tumors; i.p. vehicle), NSUN2 knockdown in combination with oxaliplatin (shNSUN2 tumors; OXA 5 mg/kg i.p. every 3 days), and oxaliplatin in combination with IKE (shCtrl tumors; OXA 5 mg/kg i.p. every 3 days and IKE 30 mg/kg i.p. daily). Vehicle injections were matched to the corresponding treatment schedule. After 14 days of treatment, mice were euthanized and tumors were collected for imaging and weighing; MDA was measured using a Lipid Peroxidation MDA Assay Kit (Beyotime, China) according to the manufacturer’s instructions.

### 2.23 Statistical analysis

Data are presented as mean ± SD. Comparisons were performed using Student’s t-test, one-way ANOVA or two-way ANOVA with multiple-comparisons correction where applicable (GraphPad Prism 9). A p-value <0.05 was considered statistically significant (*p < 0.05; **p < 0.01; ***p < 0.001; ****p < 0.0001).

## 3 Results

### 3.1 Elevated NSUN2 expression predicts poor prognosis in CRC

**FIGURE 1:**
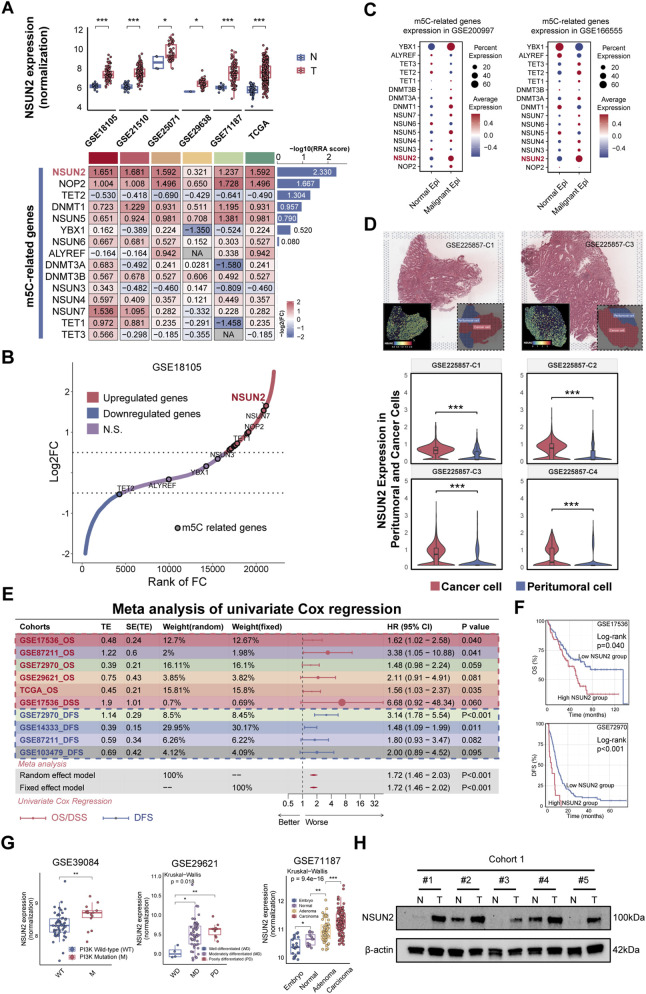
NSUN2 is overexpressed in CRC and associated with poor prognosis. **(A)** Heatmap of differential mRNA expression of m5C-related genes between CRC tumor tissues (T) and adjacent normal tissues (N) across multiple bulk transcriptomic cohorts. Bar plot showing the rank order of each gene. **(B)** Volcano plot of differentially expressed genes (DEGs) between CRC tumor and adjacent normal tissues in GSE18105. **(C)** Dot heatmap of m5C-related gene expression in malignant versus normal epithelial cells. Dot size indicates the fraction of expressing cells, color indicates average normalized expression (Epi, epithelial). **(D)** H&E staining and spatial heatmaps showing NSUN2 distribution in tumor and peritumoral regions. **(E)** Meta-analysis of univariate Cox regression of NSUN2 expression across multiple CRC bulk transcriptomic cohorts. **(F)** Kaplan–Meier survival curves for overall survival (OS) or disease-free survival (DFS) in CRC patients stratified by NSUN2 high and low expression across multiple CRC bulk transcriptomic cohorts. **(G)** Box plots of NSUN2 expression in different CRC sample groups. **(H)** Western blotting of NSUN2 protein in paired CRC tumor (T) and adjacent normal (N) tissues *p < 0.05; **p < 0.01; ***p < 0.001.

To explore the role of m5C modification in CRC, the expression of major m5C-related genes was examined across multi-omics datasets (Supplementary Table S5). Most genes exhibited differential expression between CRC tumor tissues and matched normal tissues. Robust rank aggregation (RRA) analysis identified NSUN2 as the top-ranked gene, displaying consistent overexpression with marked fold changes across multiple cohorts (Figures 1A,B; Supplementary Table S6). Single-cell transcriptomics further demonstrated preferential expression of NSUN2 within epithelial cell clusters of tumor tissues, with higher expression frequency and abundance compared with other m5C-related genes (Supplementary Figure S1A; Figure 1C). Based on pathologist-annotated spatial transcriptomic data, NSUN2 showed higher expression in tumor regions than in adjacent peritumoral regions (Figure 1D; Supplementary Figure S1B), and was further upregulated in liver metastatic tumor regions compared with primary tumor regions (Wang et al., 2023) (Supplementary Figure S1C). Collectively, multi-omics analyses indicate that NSUN2 exhibits both elevated expression and notable specificity in CRC.

To assess its prognostic value, a meta-validation cohort of seven studies (n = 1,550) was constructed. A meta-analysis of Cox regression results demonstrated that NSUN2 serves as a risk factor for poor prognosis in CRC patients, with consistent effects observed across all included studies (meta-HR [95% CI]: 1.72 [1.46–2.03]) (Figure 1E; Supplementary Table S7). Importantly, after adjustment for established clinicopathological variables, including AJCC/Dukes stage, KRAS mutation status, and chemotherapy response where available, NSUN2 remained an adverse prognostic factor across cohorts (Supplementary Figure S1D). Kaplan-Meier analysis further confirmed shorter overall survival (OS) and disease-free survival (DFS) in NSUN2-high groups (Figure 1F; Supplementary Figure S1E). Notably, NSUN2 was enriched in poorly differentiated tumors, associated with PI3K mutations, and exhibited a stage-dependent gradient across the normal–adenoma–carcinoma sequence (Figure 1G). Consistent with a more aggressive clinical phenotype, NSUN2 expression was higher in metastatic lesions than in primary tumors (Supplementary Figure S1F) and was significantly associated with greater local invasion depth (T), lymph node involvement (N), distant metastatic status (M), and overall stage in TCGA (Supplementary Figures S1G–J), collectively supporting a clinical association between NSUN2 expression and tumor invasion and metastasis. Elevated NSUN2 protein expression was also validated in freshly collected CRC tissues (Figure 1H). These results suggest that NSUN2 is frequently overexpressed and serves as an unfavorable prognostic biomarker in CRC.

### 3.2 Targeting NSUN2 suppresses CRC progression and enhances oxaliplatin sensitivity

**FIGURE 2:**
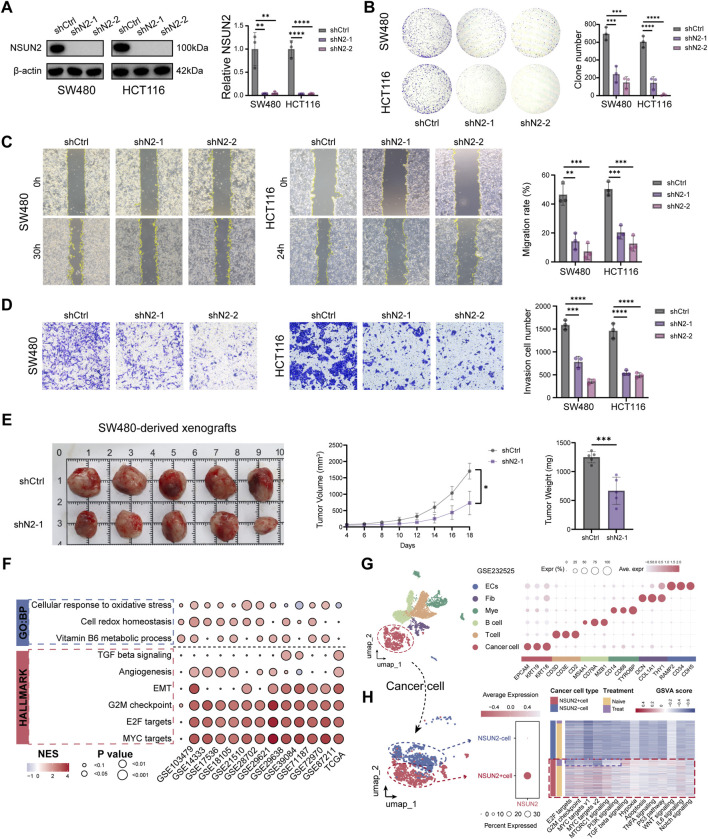
Knockdown of NSUN2 inhibits CRC progression *in vitro* and *in vivo*. **(A)** NSUN2 knockdown efficiency in SW480 and HCT116 cells assessed by Western blotting. **(B)** Proliferation of NSUN2 knockdown and control cells by colony formation assay. **(C)** Migration ability of NSUN2 knockdown and control cells detected by wound healing assays. **(D)** Invasion ability of NSUN2 knockdown and control cells detected by transwell assays. **(E)** Tumor volume and weight of xenografts derived from NSUN2 knockdown and control cells. **(F)** Dot heatmap of pathway enrichment analysis based on DEGs between high and low NSUN2 expression groups (dot size, adjusted p-value; color, normalized enrichment score [NES]). **(G)** UMAP visualization of the CRC tissue atlas and dot heatmap of reference marker expression across cell types (dot size, fraction of expressing cells; color, average normalized expression levels). **(H)** GSVA showing activity of tumor-promoting pathways in cancer cell clusters stratified by NSUN2 expression and exposure to FOLFOX-Bevacizumab treatment *p < 0.05; **p < 0.01; ***p < 0.001; ****p < 0.0001.

To examine the functional role of NSUN2, SW480 and HCT116 cells were transduced with lentiviral shRNAs, resulting in >90% knockdown efficiency as confirmed by Western blotting (Figure 2A). NSUN2 depletion significantly impaired colony formation (Figure 2B), migration (Figure 2C), and invasion (Figure 2D). In xenograft models, tumors derived from NSUN2-deficient SW480 cells showed reduced growth and weight compared with controls (Figure 2E). Collectively, these findings indicate that targeting NSUN2 impairs CRC progression. Transcriptomic profiling of NSUN2-high versus NSUN2-low tumors revealed significant enrichment of proliferation-associated programs, including MYC targets, E2F targets, and the G2M checkpoint, together with invasion-related signatures such as epithelial–mesenchymal transition, angiogenesis, and TGF-β signaling (Figure 2F; Supplementary Figure S2; Supplementary Table S8). In single-cell transcriptomic analyses, cancer cell clusters were stratified into NSUN2-positive and NSUN2-negative populations (Figure 2G). GSVA revealed that cancer cell clusters stratified by NSUN2 expression and chemotherapy exposure exhibited distinct enrichment patterns of tumor-promoting pathways. Notably, NSUN2-positive cells were preferentially enriched in oncogenic pathways, including cell cycle regulation and DNA damage repair, consistent with the bulk transcriptomic GSEA results (Figure 2H).

**FIGURE 3:**
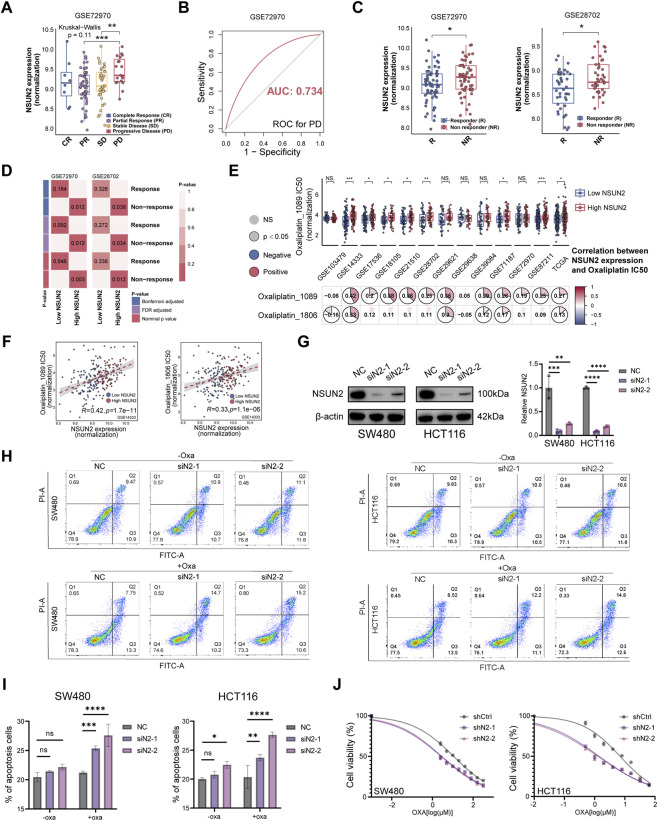
NSUN2 promotes oxaliplatin resistance in CRC. **(A)** Box plots of NSUN2 expression in CRC patients with different therapeutic responses (CR, complete response; PR, partial response; SD, stable disease; PD, progressive disease) in GSE72970. **(B)** ROC analysis of NSUN2 expression predicting PD in GSE72970. **(C)** Box plots of NSUN2 expression in responders (R) and non-responders (NR) in GSE72970 and GSE28702. **(D)** SubMap analysis predicting chemotherapy response of high- and low-NSUN2 subgroups in GSE72970 and GSE28702. **(E)** Correlation between NSUN2 expression and oxaliplatin IC50 across CRC cohorts (point size, sample size; color, correlation strength). **(F)** Scatter plots of GSE14333 showing positive correlations between NSUN2 expression and oxaliplatin IC50. **(G)** NSUN2 knockdown efficiency in SW480 and HCT116 cells assessed by Western blotting. **(H,I)** Flow cytometry analysis of apoptosis in SW480 and HCT116 cells after NSUN2 knockdown and oxaliplatin treatment. **(J)** Cell viability assays assessing the effect of NSUN2 on oxaliplatin sensitivity *p < 0.05; **p < 0.01; ***p < 0.001; ****p < 0.0001.

Given that NSUN2 is primarily expressed in malignant epithelial cells and is positively correlated with poor prognosis, we further investigated its potential association with oxaliplatin resistance. In GSE72970, progressive disease (PD) cases displayed significantly higher NSUN2 levels than responders (Figure 3A). ROC analysis demonstrated robust performance of NSUN2 in predicting PD, with an AUC of 0.734 (Figure 3B). Consistently, across both the GSE72970 and GSE28702 cohorts, non-responders to oxaliplatin-based chemotherapy showed elevated NSUN2 expression relative to responders (Figure 3C). The results of the “SubMap” algorithm indicate that there are statistically significant commonalities between the groups based on the expression level of NSUN2 and the groups based on chemotherapy response (Figure 3D). We next assessed the association between NSUN2 expression and oxaliplatin sensitivity across multiple CRC bulk transcriptomic cohorts. NSUN2 expression was positively correlated with oxaliplatin IC50 values, and tumors with high NSUN2 expression consistently exhibited reduced chemosensitivity (Figure 3E; Supplementary Tables S9, S10). In the GSE14333 cohort, NSUN2 expression was positively correlated with oxaliplatin IC50 values (Oxaliplatin_1806: R = 0.33, p = 1.1 × 10^−6^; Oxaliplatin_1089: R = 0.42, p = 1.7 × 10^−11^) (Figure 3F).

To experimentally validate these findings, we assessed apoptosis in CRC cells following oxaliplatin treatment. Due to GFP interference from shRNA vectors in FITC-based assays, siRNA-mediated NSUN2 knockdown was employed, resulting in over 70% reduction in protein expression (Figure 3G). Flow cytometry revealed that siRNA-mediated knockdown of NSUN2 enhanced oxaliplatin-induced apoptosis (Figures 3H,I), and CCK-8 assays showed that NSUN2 depletion markedly increased cellular sensitivity to oxaliplatin (Figure 3J). Collectively, these findings demonstrate that high NSUN2 expression is associated with CRC progression and oxaliplatin resistance, whereas its inhibition suppresses malignant phenotypes and sensitizes tumor cells to treatment.

### 3.3 NSUN2 inhibition sensitizes CRC cells to ferroptosis

**FIGURE 4:**
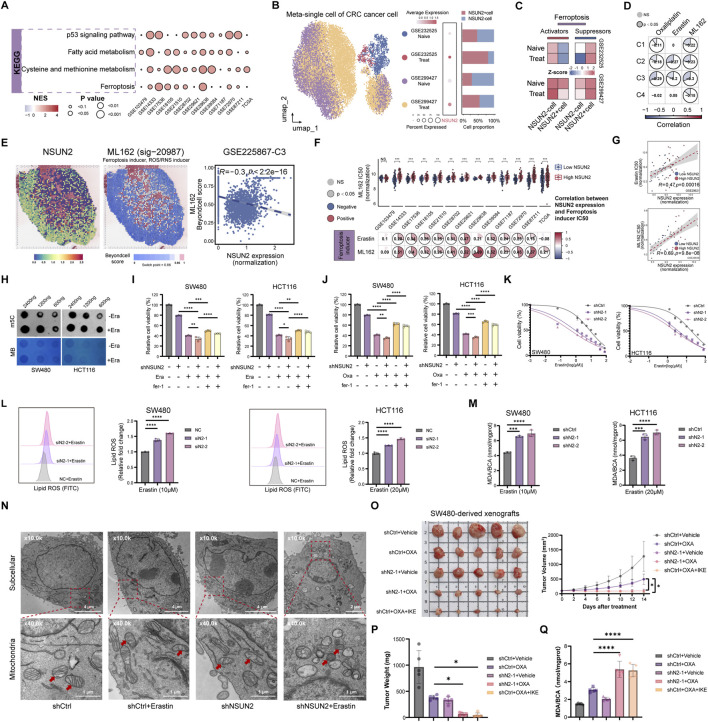
NSUN2 knockdown induces ferroptosis in CRC. **(A)** KEGG enrichment analysis based on DEGs between high and low NSUN2 expression groups (dot size, adjusted p-value; color, normalized enrichment score [NES]). **(B)** UMAP visualization of meta-single-cell CRC datasets showing NSUN2 expression in naïve and treated samples; dot heatmap of NSUN2 expression across treated clusters; bar plot of NSUN2-positive cell proportions. **(C)** Heatmap of ferroptosis-related pathway activity between NSUN2-positive and NSUN2-negative cancer cell clusters. **(D)** Correlations between NSUN2 expression and Beyondcell score (BCS; higher BCS indicates greater predicted drug sensitivity) for oxaliplatin and ferroptosis inducers (erastin, ML162) across four CRC spatial transcriptomic samples in GSE225867. **(E)** Spatial distribution of NSUN2 expression and ML162 sensitivity in GSE225867-C3; scatter plots showing the correlation between NSUN2 expression and BCS. **(F)** Correlation between NSUN2 expression and IC50 values of ferroptosis inducers in bulk CRC samples. **(G)** Scatter plots showing correlations between NSUN2 expression and erastin/ML162 IC50 in GSE29621 and GSE29638. **(H)** Dot blot of global RNA m5C modification in CRC cells with or without erastin treatment; methylene blue (MB) staining as loading control. **(I,J)** Cell viability assays showing the effect of Ferrostatin-1 (Fer-1) on erastin **(I)** and oxaliplatin **(J)** cytotoxicity in NSUN2 knockdown cells. **(K)** Cell viability assays showing the effect of NSUN2 on erastin sensitivity. **(L)** Flow cytometry of lipid ROS in CRC cells after NSUN2 silencing and erastin treatment. **(M)** Malondialdehyde (MDA) levels in control and NSUN2 knockdown CRC cells after 24 h erastin treatment. **(N)** Transmission electron microscopy (TEM) of mitochondrial morphology in NSUN2 knockdown and control cells. **(O)** Photograph of excised SW480 xenografts and tumor growth kinetics during the 14-day treatment period. **(P)** Tumor weights of excised SW480 xenografts at the end of the 14-day treatment period. **(Q)** Tumor lipid peroxidation assessed by malondialdehyde (MDA) quantification in xenografts collected at the end of treatment *p < 0.05; **p < 0.01; ***p < 0.001; ****p < 0.0001.

Building on the KEGG enrichment results (Figure 4A; Supplementary Table S8) and the observed association with oxaliplatin sensitivity, we next investigated whether NSUN2 promotes CRC progression and therapy resistance through ferroptosis-related mechanisms. In oxaliplatin-treated single-cell datasets, NSUN2-positive cells were enriched in resistant clusters (Figure 4B). Concurrently, ssGSEA revealed ferroptosis-suppressive activity in NSUN2-positive cell clusters, whereas NSUN2-negative clusters exhibited ferroptosis-promoting activity. These differences were more pronounced after oxaliplatin treatment (Figure 4C; Supplementary Tables S11, S12). Spatial transcriptomics further demonstrated a significant negative spatial correlation between NSUN2 expression levels and sensitivity to classical ferroptosis inducers (such as erastin and ML162) in independent CRC samples (Figures 4D,E; Supplementary Table S13). This finding was corroborated in multiple bulk transcriptomic CRC datasets, where NSUN2 expression showed a significant positive correlation with the IC50 values of various ferroptosis inducers (Figures 4F,G; Supplementary Tables S9, S10). Collectively, these multi-omics analyses indicate that elevated NSUN2 expression is associated with reduced ferroptosis sensitivity.

As NSUN2 functions as an RNA m5C methyltransferase, we next evaluated whether ferroptosis sensitivity was influenced by RNA methylation status. Dot blot analysis showed increased global RNA m5C levels after erastin treatment (Figure 4H). Pharmacological inhibition with Ferrostatin-1 (Fer-1) significantly attenuated both oxaliplatin- and erastin-induced cytotoxicity in CRC cells, indicating that ferroptosis constitutes a major cell death pathway in this setting (Figures 4I,J). NSUN2 knockdown markedly sensitized CRC cells to ferroptosis, evidenced by reduced viability (Figure 4K), elevated lipid ROS (Figure 4L), and increased malondialdehyde (MDA) production (Figure 4M). Transmission electron microscopy (TEM) also revealed classical ultrastructural changes observed during ferroptosis—including condensed mitochondrial membranes, cristae disruption, and outer membrane rupture—in NSUN2-deficient CRC cells, both at baseline and following erastin treatment (Figure 4N). To further validate the *in vivo* relevance of NSUN2-mediated ferroptosis resistance and oxaliplatin responsiveness, we performed nude mouse xenograft experiments evaluating oxaliplatin alone and oxaliplatin combined with the ferroptosis inducer imidazole ketone erastin (IKE). Tumor growth was more effectively suppressed by oxaliplatin in NSUN2-depleted xenografts than by oxaliplatin alone (Figures 4O,P), supporting *in vivo* chemosensitization upon NSUN2 depletion. In parallel, oxaliplatin combined with IKE further reduced tumor burden compared with oxaliplatin monotherapy (Figures 4O,P), highlighting the potential translational value of ferroptosis-promoting strategies to enhance oxaliplatin response. Mechanistically, tumor malondialdehyde (MDA) levels were increased in oxaliplatin-treated NSUN2-depleted tumors and in tumors treated with oxaliplatin combined with IKE, relative to tumors treated with oxaliplatin alone (Figure 4Q). These findings support enhanced lipid peroxidation and elevated ferroptotic stress *in vivo*.

Collectively, these data demonstrate that NSUN2 inhibition enhances ferroptosis sensitivity in CRC cells, whereas elevated NSUN2 expression is linked to ferroptosis resistance and reduced oxaliplatin efficacy.

### 3.4 NSUN2-mediated m5C modification stabilizes DHODH mRNA

**FIGURE 5:**
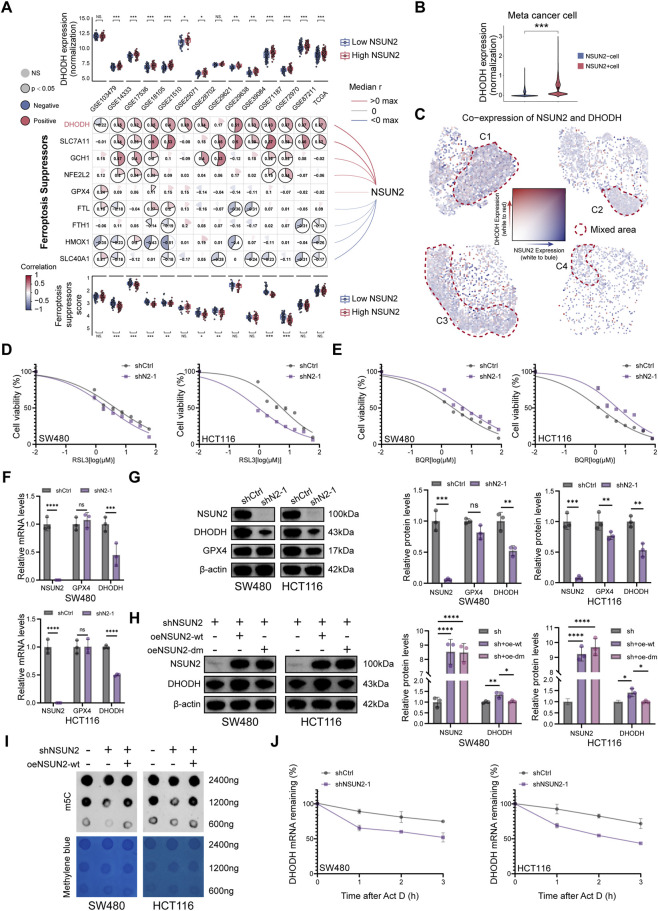
NSUN2 regulates ferroptosis resistance in CRC through stabilization of DHODH mRNA. **(A)** Correlations between NSUN2 and major ferroptosis-related genes across CRC cohorts. **(B)** Violin plot showing preferential DHODH expression in NSUN2-positive malignant epithelial populations at the single-cell level. **(C)** Co-expression heatmap showing spatial overlap between NSUN2 and DHODH. **(D,E)** Cell viability assays of NSUN2 knockdown and control cells treated with GPX4 inhibitor RSL3 **(D)** or DHODH inhibitor Brequinar (BQR) **(E). (F)** qRT-PCR analysis of GPX4 and DHODH mRNA in NSUN2 knockdown and control cells. **(G)** Western blotting of GPX4 and DHODH protein in NSUN2 knockdown and control cells. **(H)** Western blotting of DHODH protein in NSUN2-silenced cells re-expressing wild-type or enzymatically inactive NSUN2 mutants (C271A, C321A). **(I)** Dot blot of total RNA m5C levels in control cells, shNSUN2 cells, and shNSUN2 cells re-expressing wild-type NSUN2. **(J)** DHODH mRNA decay measured after actinomycin D treatment *p < 0.05; **p < 0.01; ***p < 0.001; ****p < 0.0001.

To further dissect the molecular basis underlying NSUN2-mediated ferroptosis resistance, we systematically interrogated the expression correlation between NSUN2 and major ferroptosis-related genes across multiple CRC bulk transcriptomic cohorts. Meta-cohort analyses identified dihydroorotate dehydrogenase (DHODH) as the ferroptosis suppressor most consistently associated with NSUN2 expression (Figure 5A; Supplementary Tables S14, S15). While NSUN2 expression showed little association with GPX4 or other ferroptosis-related genes, DHODH expression was markedly elevated in NSUN2-high CRC samples, suggesting a unique regulatory relationship. At the single-cell level, DHODH expression was preferentially enriched in malignant epithelial cells with high NSUN2 expression, whereas low-NSUN2 populations exhibited weaker DHODH expression (Figure 5B). Furthermore, joint visualization of NSUN2 and DHODH co-expression patterns in spatial transcriptomic samples revealed significant spatial co-localization within specific tumor subclusters, implying overlapping enrichment in therapy-resistant epithelial regions (Figure 5C). These findings pinpoint DHODH as a putative downstream effector selectively regulated by NSUN2.

Functionally, NSUN2 knockdown sensitized cells to RSL3, a GPX4 inhibitor, but conferred relative resistance to the DHODH inhibitor Brequinar (BQR) (Figures 5D,E). This indicates that NSUN2 differentially modulates GPX4-and DHODH-mediated ferroptosis. qRT-PCR and WB further showed that NSUN2 knockdown markedly suppressed DHODH expression at both mRNA and protein levels, while GPX4 expression remained unaffected (Figures 5F,G). Mechanistically, only wild-type NSUN2, but not catalytically inactive mutants (C271A, C321A), restored DHODH expression in NSUN2-deficient CRC cells (Figure 5H). Global RNA m5C levels were reduced by NSUN2 knockdown and rescued by wild-type NSUN2 (Figure 5I). RNA decay assays further showed accelerated DHODH mRNA degradation in the absence of NSUN2 (Figure 5J). Collectively, these results indicate that NSUN2 stabilizes DHODH mRNA through m5C modification.

### 3.5 NSUN2–DHODH axis confers oxaliplatin resistance through ferroptosis suppression

**FIGURE 6:**
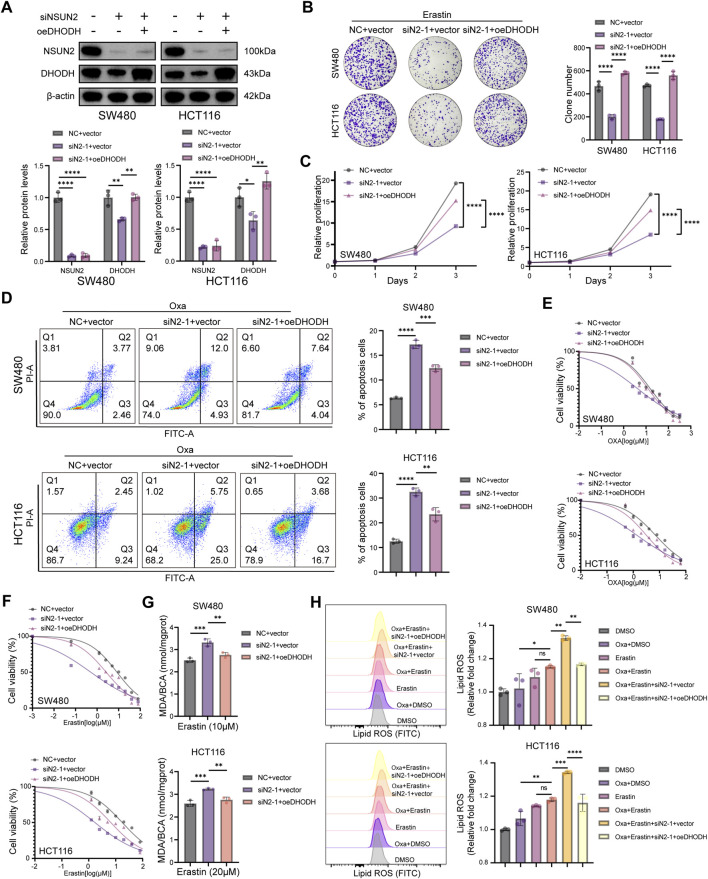
NSUN2 promotes oxaliplatin resistance via DHODH-mediated ferroptosis suppression. **(A)** Western blotting of NSUN2 and DHODH in NSUN2 knockdown CRC cells. **(B,C)** Colony formation **(B)** and proliferation **(C)** assays of NSUN2 knockdown cells treated with erastin with or without DHODH overexpression. **(D)** Flow cytometry of apoptosis in NSUN2 knockdown cells with or without DHODH overexpression after oxaliplatin treatment. **(E,F)** Cell viability assays of NSUN2 knockdown cells with or without DHODH overexpression treated with erastin **(E)** or oxaliplatin **(F)**. **(G,H)** Quantification of MDA levels **(G)** and flow cytometric analysis **(H)** of lipid ROS in NSUN2 knockdown cells with or without DHODH overexpression *p < 0.05; **p < 0.01; ***p < 0.001; ****p < 0.0001.

To determine whether DHODH mediates the ferroptosis resistance and oxaliplatin tolerance conferred by NSUN2, we ectopically expressed DHODH in NSUN2-silenced SW480 and HCT116 cells. Western blotting confirmed efficient restoration of DHODH expression following its overexpression (Figure 6A). Functionally, colony formation and CCK-8 assays demonstrated that NSUN2 knockdown markedly enhanced erastin-induced growth inhibition, whereas DHODH overexpression largely reversed these effects (Figures 6B,C). Consistent with this, flow cytometry analysis revealed that DHODH overexpression attenuated oxaliplatin-induced apoptosis in NSUN2-deficient cells (Figure 6D). Drug sensitivity assays further indicated that DHODH overexpression counteracted the enhanced susceptibility of NSUN2-silenced cells to both erastin and oxaliplatin (Figures 6E,F). Mechanistically, DHODH overexpression markedly attenuated MDA production and lipid ROS accumulation induced by NSUN2 knockdown (Figures 6G,H), suggesting that the NSUN2–DHODH axis promotes ferroptosis resistance by suppressing lipid peroxidation.

**FIGURE 7:**
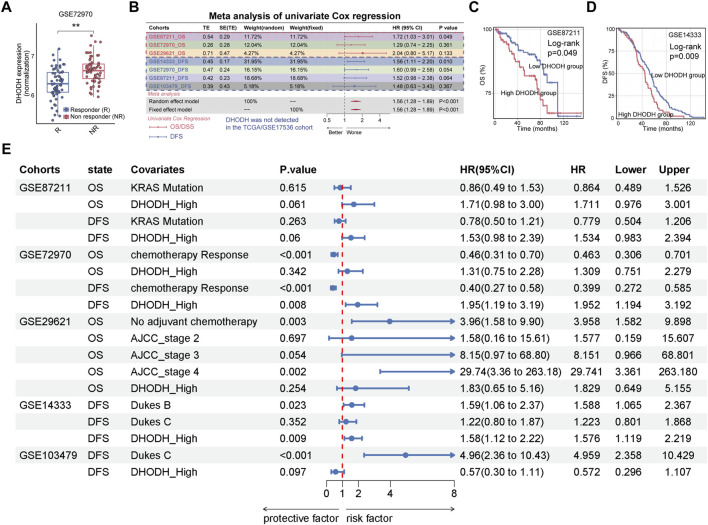
Clinical relevance of DHODH expression in colorectal cancer. **(A)** Box plot of DHODH expression in responders (R) and non-responders (NR) in GSE72970. **(B)** Meta-analysis of univariate Cox regression of DHODH expression across multiple CRC bulk transcriptomic cohorts. **(C,D)** Kaplan–Meier survival curves for OS (GSE87211) **(C)** and DFS (GSE14333) **(D)** according to DHODH expression. **(E)** Multivariable Cox regression analyses of DHODH expression across CRC cohorts **p < 0.01.

To assess the clinical relevance, we next interrogated public CRC transcriptomic datasets. High DHODH expression was associated with poor chemotherapy response in GSE72970 (Figure 7A). Meta-analysis of Cox regression across multiple CRC bulk transcriptomic datasets confirmed its prognostic significance (Figure 7B; Supplementary Table S16), and Kaplan-Meier analyses of GSE14333 and GSE87211 demonstrated worse survival in DHODH-high patients (Figures 7C,D). Importantly, after adjustment for tumor stage, KRAS mutation status, and chemotherapy response where available, DHODH remained an independent adverse prognostic factor in multivariable Cox analyses (Figure 7E). Together, these results establish the NSUN2–DHODH axis as a mediator of ferroptosis suppression and oxaliplatin resistance in CRC.

## 4 Discussion

Numerous chemotherapeutic agents can trigger ferroptosis, and disruption of this process is increasingly recognized as a driver of chemoresistance and treatment failure (Zhang et al., 2022). Accordingly, pharmacological or genetic modulation of ferroptosis has been shown to overcome resistance to chemotherapy (Zhou et al., 2024; Ascenzi et al., 2024). For instance, the iron chelators deferoxamine (DFO) and deferiprone (DFP) can reverse temozolomide (TMZ) resistance in glioblastoma (GBM) by disrupting iron-dependent pathways critical for tumor cell survival and repair (Amereh et al., 2025). In recent years, lipid ROS imbalance–mediated ferroptosis has been increasingly implicated in tumor drug resistance (Kim et al., 2023; Wang et al., 2024; de Laat et al., 2024). For example, ubiquitin-mediated degradation of the lipid peroxidation–associated enzyme acyl-CoA synthetase long-chain family member 4 (ACSL4) suppresses ferroptosis and promotes oxaliplatin resistance in CRC (Zeng et al., 2023). Similarly, inhibition of keratin 17 (K17) reduces the stability of dihydroorotate dehydrogenase (DHODH), thereby promoting ferroptosis and reversing gemcitabine resistance in pancreatic ductal adenocarcinoma (PDAC) (Lyu et al., 2024). Moreover, combining ferroptosis inducers with conventional chemotherapeutics has demonstrated significant synergistic antitumor effects, representing a potential therapeutic strategy across multiple tumor types (Han et al., 2022; Pei et al., 2025; Zhao et al., 2023). For example, the ferroptosis inducer RSL3 markedly enhances the efficacy of TMZ against GBM (Miao et al., 2024). Several clinically approved drugs, such as sorafenib and cisplatin, have also been shown to trigger ferroptosis (Yuan et al., 2022; Liu et al., 2023; Lin et al., 2023; Guo et al., 2024; Chen et al., 2021). Nevertheless, the mechanisms by which tumor cells restrain ferroptosis to acquire chemoresistance remain incompletely defined.

Here, we identified NSUN2 as a progression-associated gene in CRC. As a key RNA methyltransferase catalyzing 5-methylcytosine (m5C) formation, NSUN2 has drawn increasing attention for its roles in mRNA modification and disease progression. While previous research has predominantly focused on the oncogenic roles of NSUN2, its function in regulating cell death and chemoresistance in CRC remains largely unexplored (Chen et al., 2024; Zou et al., 2024). Integrating multi-omics datasets with validation in patient samples, we observed NSUN2 upregulation in colorectal cancer and enrichment in clinically aggressive subsets. Functionally, NSUN2 depletion attenuated malignant phenotypes and reduced tumor growth *in vivo*, supporting a contributory role in disease progression; however, orthotopic and/or experimental metastasis models will be required to directly evaluate the causal role of NSUN2 in metastatic dissemination and will be prioritized in future studies. Consistent with its association with unfavorable chemotherapy outcomes, NSUN2 knockdown increased oxaliplatin sensitivity *in vitro*. Importantly, xenograft experiments further showed that oxaliplatin achieved greater tumor suppression under NSUN2 depletion than in control tumors, providing *in vivo* support that NSUN2 limits oxaliplatin responsiveness.

Mechanistically, pathway analyses suggested involvement of oxidative stress-related programs, prompting us to test ferroptosis as a functional mediator. Using ferrostatin-1, we verified that ferroptosis contributes substantially to oxaliplatin-associated cytotoxicity in our models. NSUN2 knockdown increased lipid peroxidation markers and induced ultrastructural mitochondrial changes consistent with ferroptotic stress, supporting the concept that NSUN2 promotes chemoresistance, at least in part, by constraining lipid peroxidation–driven cell death. Consistently, tumor MDA levels were higher in oxaliplatin-treated tumors under NSUN2 depletion than in oxaliplatin-treated control tumors, which is consistent with enhanced lipid peroxidation and elevated ferroptotic stress *in vivo*. In parallel, combining oxaliplatin with the ferroptosis inducer IKE further reduced tumor burden compared with oxaliplatin alone, supporting the feasibility of enhancing oxaliplatin activity through ferroptosis-promoting strategies in preclinical CRC models.

**FIGURE 8:**
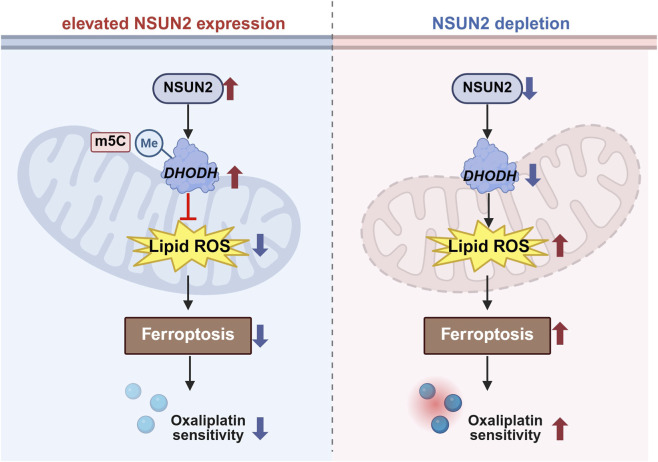
Schematic illustration of NSUN2 function in CRC. Proposed model of NSUN2-mediated m5C modification of DHODH mRNA promoting ferroptosis resistance and oxaliplatin resistance in CRC. Created with BioRender.com.

We further focused on how NSUN2 interfaces with ferroptosis regulators. Glutathione peroxidase 4 (GPX4) and ACSL4 are key regulators of lipid peroxidation in ferroptosis (Yang et al., 2014; Doll et al., 2017). Notably, recent evidence has identified DHODH, an enzyme located on the outer surface of the inner mitochondrial membrane, as a critical ferroptosis suppressor (Mao et al., 2021). DHODH functions by reducing ubiquinone (CoQ) to ubiquinol (CoQH2), thereby mitigating mitochondrial lipid peroxidation in a GPX4-independent manner (Cao et al., 2025). Inactivation of DHODH induces widespread mitochondrial lipid peroxidation and ferroptosis in cancer cells (Mao et al., 2021). Our bioinformatics and experimental data revealed significant upregulation of DHODH in CRC, with a positive correlation to NSUN2 expression. Mechanistically, NSUN2 depletion reduced DHODH expression, which augmented lipid peroxidation and sensitized CRC cells to ferroptosis. Overexpression of DHODH partially rescued the ferroptosis effects induced by NSUN2 knockdown. These findings indicate that NSUN2 modulates oxaliplatin sensitivity by stabilizing DHODH mRNA through m5C modification, thereby limiting lipid peroxidation and ferroptosis (Figure 8). Several considerations refine the mechanistic and translational interpretation. First, while our data show a strong correlation between NSUN2 and DHODH expression, the causal hierarchy between NSUN2-mediated m5C modification and DHODH mRNA stabilization could be further strengthened. Our study demonstrated that wild-type NSUN2, but not enzymatic-dead mutants (C271A, C321A), restored DHODH expression, and that NSUN2 depletion led to accelerated DHODH mRNA decay. These findings are consistent with a mechanism by which NSUN2 stabilizes DHODH mRNA through m5C modification, and we believe they provide a robust foundation for future mechanistic investigations. Nonetheless, we acknowledge that site-specific mapping of m5C modifications remains an important future direction to refine our understanding of NSUN2-dependent m5C deposition on DHODH transcripts, and we will prioritize this in follow-up studies. Regarding the broader ferroptosis regulatory landscape, while our study emphasizes the role of DHODH in ferroptosis suppression independent of GPX4, we have further explored potential interactions between NSUN2 and other ferroptosis-related pathways. Using bulk transcriptomic data from colorectal cancer cohorts, we observed that NSUN2 expression is weakly correlated with other ferroptosis suppressors such as GCH1, FSP1, and GSTP1, but these correlations were not as strong as the one with DHODH (Supplementary Figure S3; Supplementary Tables S17, S18). This suggests that DHODH represents the dominant ferroptosis suppressor associated with NSUN2 expression in colorectal cancer, whereas other pathways may be regulated in a more context-dependent manner. However, with the growing understanding of ferroptosis pathways, an increasing number of studies have highlighted the complex crosstalk between these pathways (Doll et al., 2019; Kim et al., 2022). For instance, the FSP1 and GPX4 pathways exhibit significant complementary and compensatory activation in the regulation of ferroptosis (Wu et al., 2026; Palma et al., 2026). Therefore, the interplay between these pathways in mediating ferroptosis resistance in colorectal cancer warrants further investigation. In light of this, future studies should explore potential cross-talk between NSUN2 and other ferroptosis-related pathways, such as the FSP1–CoQ10 and GCH1–BH4 axes, to clarify the specificity and breadth of NSUN2-mediated ferroptosis regulation in colorectal cancer. Finally, although no clinically approved NSUN2 inhibitors are currently available, emerging NSUN2-targeting chemical probes provide an initial framework to test the therapeutic premise that NSUN2 inhibition may enhance oxaliplatin efficacy across independent preclinical colorectal cancer models (Chen et al., 2024); however, the translational potential will require careful evaluation of on-target systemic tolerability given the broad roles of RNA methylation in normal tissue homeostasis.

In summary, our findings delineate an NSUN2–DHODH regulatory connection that restrains lipid peroxidation and contributes to oxaliplatin resistance in colorectal cancer. They provide a mechanistically grounded rationale for enhancing chemotherapy response through ferroptosis-promoting strategies.

## 5 Conclusion

We identified NSUN2 as a progression-associated gene in CRC and showed that its depletion sensitizes CRC cells to oxaliplatin by inducing ferroptosis. Mechanistically, NSUN2 stabilizes DHODH mRNA via m5C modification, thereby blocking lipid peroxidation and ferroptosis. These findings highlight NSUN2 as a potential therapeutic target to overcome chemoresistance in CRC and support further exploration of ferroptosis-based therapeutic strategies.

## Data Availability

The datasets presented in this study can be found in online repositories. The names of the repository/repositories and accession number(s) can be found in the article/Supplementary Material.
