# Supplementary material for: Targeting the NSUN2–DHODH axis reverses ferroptosis resistance and oxaliplatin resistance in colorectal cancer
Source: Front Pharmacol. 2026 Feb 23;17:1739981. doi: 10.3389/fphar.2026.1739981 (PMC12968206; doi:10.3389/fphar.2026.1739981)
Supplement: Supplementary file 1 [file DataSheet1.pdf]

Supplementary Material

1 Supplementary Figures and Tables

1.1 Supplementary Figures

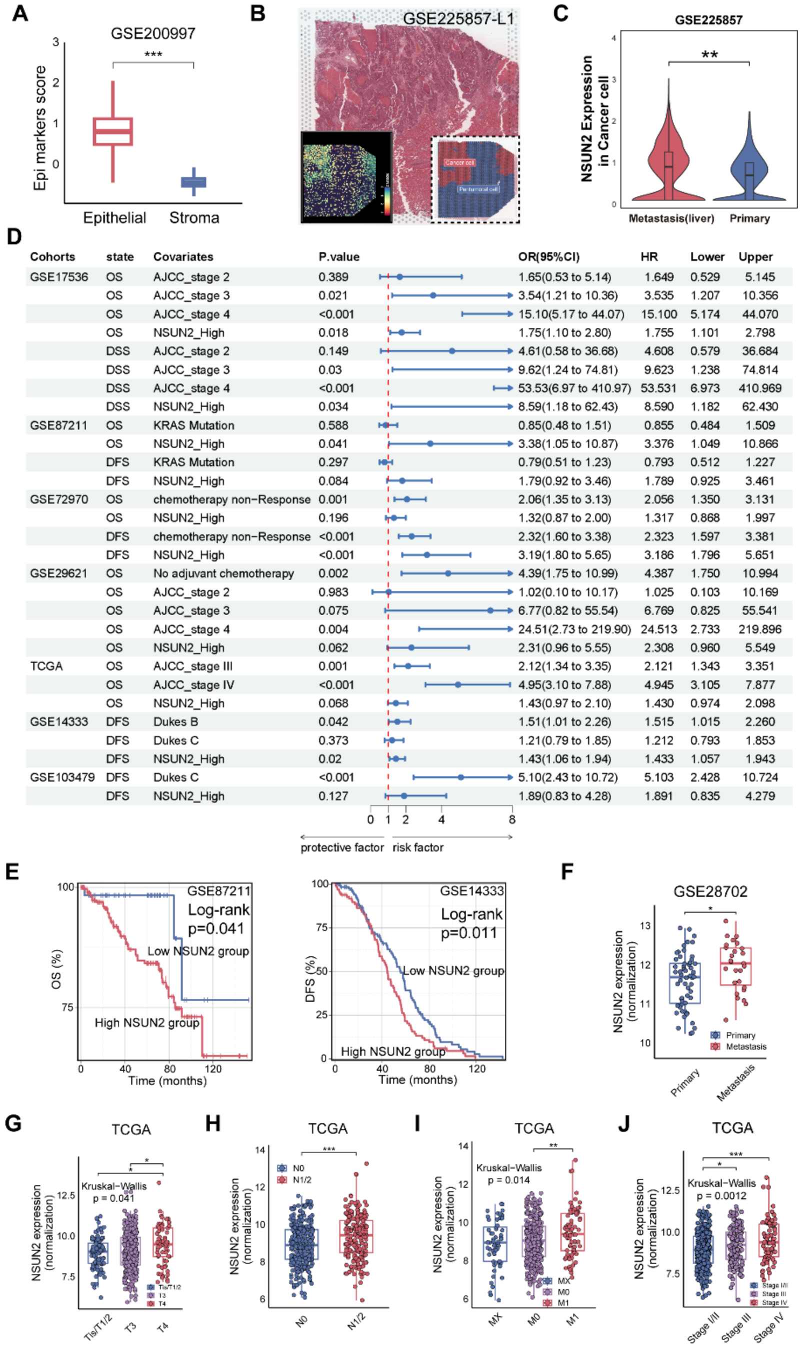

Supplementary Figure 1. Multi-omics evidence supporting NSUN2 expression and its clinical relevance in CRC. (**A**) The box plot of epithelial marker scores for manually annotated epithelial and stromal clusters in GSE200997. (**B**) Representative H&E staining and corresponding spatial

transcriptomic heatmaps showing NSUN2 distribution in liver metastasis lesions, comparing tumor and peritumoral regions. (**C**) Volcano plot showing differential expression of NSUN2 between primary CRC cancer cells and liver metastases. (**D**) Meta-analysis of multivariate Cox regression evaluating the prognostic value of NSUN2 expression across multiple CRC bulk transcriptomic cohorts. Odds ratios (ORs) and hazard ratios (HRs) with 95% confidence intervals (CIs) are shown, and covariates included in each cohort are listed (e.g., AJCC stage, Dukes stage, KRAS mutation, chemotherapy response). (**E**) Kaplan–Meier survival curves for overall survival (OS) or disease-free survival (DFS) in CRC patients stratified by NSUN2 high and low expression across multiple CRC bulk transcriptomic cohorts. (**F**) The box plot of NSUN2 expression between primary tumors and metastatic lesions in GSE28702. (**G**–**J**) Associations between NSUN2 expression and clinicopathologic features in TCGA-COAD, including tumor invasion depth (T stage) (**G**), lymph node status (N stage) (**H**), distant metastasis status (M stage) (**I**), and overall AJCC stage (**J**). Statistical tests are indicated in each panel.

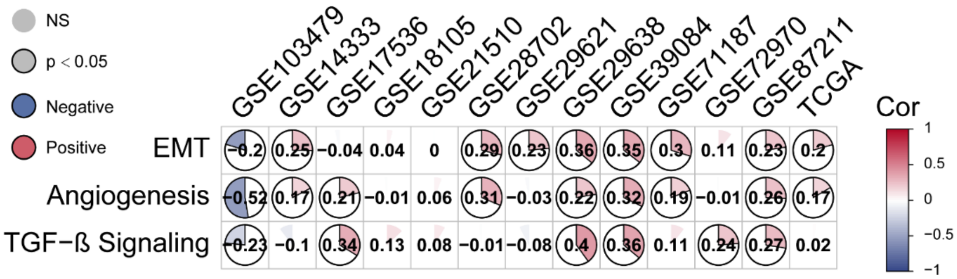

Supplementary Figure 2. Correlation between NSUN2 expression and ssGSEA pathway activity in CRC bulk transcriptomic cohorts.

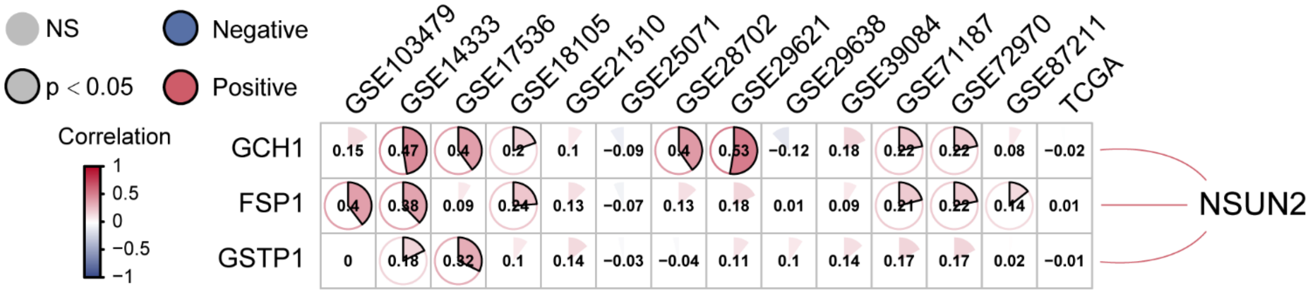

Supplementary Figure 3. Correlation between NSUN2 expression and novel ferroptosis suppressors in CRC bulk transcriptomic cohorts.

1.2 Supplementary Tables

Table S1. Summary of the bulk transcriptomics datasets

Dataset	Number of samples	Sample type	Samples with prognostic data	Tissue source	Pubmed ID
GSE103479	156	156 T	154 DFS	Human colon	30088816
GSE14333	226	226 T	226 DFS	Human colon	19996206
GSE17536	177	177 T	55 OS/177 DSS	Human colon	19914252
GSE18105	111	94 T and 17 N	/	Human colon	20162577
GSE21510	148	123 T and 25 N	/	Human colon	21270110
GSE25071	50	46 T and 4 N	/	Human colon	21716316
GSE28702	83	83 T	/	Human colon	22095227
GSE29621	65	65 T	65 OS	Human colon	22213796
GSE29638	50	50 T	/	Human colon	29900672
GSE39084	70	70 T	/	Human colon	25083765
GSE71187	189	99 T, 12 N and 78 other	/	Human colon	26325386
GSE72970	124	124 T	124 OS/124 DFS	Human colon	30863148
GSE87211	363	363 T	353 OS/353 DFS	Human colon	29119627
TCGA-COAD	491	451 T and 40 N	451 OS	Human colon	22810696

Table S2. Summary of the single-cell and spatial transcriptomics datasets

**Dataset**	**Number of patients**	**Number of samples**	**Sample type**	**Tissue source**	**Pubmed ID**
GSE166555	12	25	13 T and 12 N	25 Human colon	34409732
GSE200997	16	23	16 T and 7 N	23 Human colon	35538548
GSE232525	1	2	1 Navie and 1 FOLFOX-Bev treated	2 Human colon	37576892
GSE299427	/	4	1 Navie and 3 Oxaliplatin treated	4 HCT116 cell line	40664638
GSE225857	4	4	4 CRC(spatial)	4 Human colon	37327339

Table S3. The original cell type markers used in the references for annotation purposes

Dataset	Cell type	Markers		
GSE232525	Cancel cell	EPCAM	KRT19	KRT18
	T cell	CD3D	CD3E	CD2
	B cell	MS4A1	CD79A	MZB1
	Mye	CD14	CD68	TYROBP
	Fib	DCN	COL1A1	THY1
	ECs	RAMP2	CD34	CDH5

Table S4. Classical Colorectal epithelial markers for annotation

Dataset	Cell type	Epi_markers

GSE200997	Epithelial General Markers	EPCAM	CDH1			
	Keratin (Cellular Protein)	KRT8	KRT18	KRT19	KRT20	
	Intestinal Epithelial Differentiation Transcription Factor	CDX1	CDX2			
	Ciliated Cell Markers	MUC2	TFF3			
	Intestinal Endocrine Cells Markers	CHGA	SYNGR2			

Table S5. m5C related genes

NOP2
NSUN2
NSUN3
NSUN4
NSUN5
NSUN6
NSUN7
DNMT1
DNMT3A
DNMT3B
TET1
TET2
TET3
ALYREF
YBX1

Table S6. The results of the RRA algorithm for m5C-related genes in different bulk cohorts

Gene	GSE18105	GSE21510	GSE25071	GSE29638	GSE71187	TCGA	RRA_score	Freq

ALYREF	-0.16392709	-0.16397347	0.942096712	NA	0.337821	0.942096712	1	2
DNMT1	0.72267085	1.22938031	0.930642484	0.51133979	1.194642	0.930642484	0.110509748	5
DNMT3A	0.68330272	-0.49247408	0.241442122	0.02811813	-1.5801755	0.241442122	1	1
DNMT3B	0.56740181	0.67829397	0.527278223	0.60613771	0.4922453	0.527278223	1	4
NOP2	1.00412007	1.00849612	1.496452445	0.65011021	1.7283191	1.496452445	0.021520259	5
NSUN2	1.65079413	1.68098165	1.592338291	0.32120542	1.2365138	1.592338291	0.004674027	5
NSUN3	0.34349489	-0.48188943	-0.460237293	0.14658458	-0.8087245	-0.460237293	1	1
NSUN4	0.59747649	0.40909417	0.356925135	0.12131458	0.4487838	0.356925135	1	1
NSUN5	0.65146522	0.9240307	0.98075196	0.7083775	1.3808083	0.98075196	0.162336701	5
NSUN6	0.66657495	0.68142678	0.526861102	0.15168625	0.3030014	0.526861102	0.852012692	4
NSUN7	1.53616822	1.09468212	0.28212129	-0.33222021	0.2283548	0.28212129	1	2
TET1	0.97175817	0.88082828	0.235072938	-0.29074187	-1.4581398	0.235072938	1	2
TET2	-0.53044489	-0.41801749	-0.690318891	-0.42891583	-0.6410743	-0.490318891	0.049687337	3
TET3	0.56612971	-0.29774879	-0.18529991	-0.35468875	NA	-0.18529991	1	1
YBX1	0.16159323	-0.38920774	0.224119144	-1.35017562	-0.5236109	0.224119144	0.302683101	1

Table S7. The Cox regression results of the NSUN2 gene on prognosis in different clinical cohorts

Dataset	Gene	HR	CI_lower	CI_upper	PValue	state
TCGA	NSUN2	1.562898	1.032798	2.365081	0.034631	OS
GSE17536	NSUN2	1.623244	1.021942	2.578348	0.04018	OS

GSE29621	NSUN2	2.114654	0.910878	4.909288	0.081379	OS
GSE72970	NSUN2	1.484231	0.984627	2.237336	0.059298	OS
GSE87211	NSUN2	3.379804	1.050125	10.87783	0.041154	OS
GSE17536	NSUN2	6.682978	0.923922	48.3398	0.059892	DSS
GSE14333	NSUN2	1.477001	1.094392	1.993374	0.010784	DFS
GSE87211	NSUN2	1.795548	0.927993	3.474156	0.082202	DFS
GSE72970	NSUN2	3.141674	1.783179	5.535123	7.45E-05	DFS
GSE103479	NSUN2	1.999637	0.885372	4.516233	0.095499	DFS

Table S8. The differential gene enrichment results of the high and low NSUN2 subgroups in different bulk cohorts

Dataset	Pathway	NES	neg_log10_padj	Category	padj
GSE103479	Fatty acid metabolism	1.43341	1.428023	KEGG	<0.05
GSE14333	Fatty acid metabolism	1.873776	4.086472	KEGG	<0.001
GSE17536	Fatty acid metabolism	1.07724	0.485201	KEGG	>0.1
GSE18105	Fatty acid metabolism	1.644848	2.931814	KEGG	<0.01
GSE21510	Fatty acid metabolism	1.556142	2.214515	KEGG	<0.01
GSE28702	Fatty acid metabolism	1.386971	1.204723	KEGG	<0.1
GSE29621	Fatty acid metabolism	1.358942	1.303045	KEGG	<0.05
GSE29638	Fatty acid metabolism	1.77094	2.707953	KEGG	<0.01

GSE39084	Fatty acid metabolism	1.511635	1.846942	KEGG	<0.05
GSE71187	Fatty acid metabolism	1.377238	1.358464	KEGG	<0.05
GSE72970	Fatty acid metabolism	1.658407	2.52871	KEGG	<0.01
GSE87211	Fatty acid metabolism	0.990394	0.333215	KEGG	>0.1
TCGA	Fatty acid metabolism	1.299919	1.057295	KEGG	<0.1
GSE103479	p53 signaling pathway	1.200211	0.74999	KEGG	>0.1
GSE14333	p53 signaling pathway	1.876498	4.589126	KEGG	<0.001
GSE17536	p53 signaling pathway	1.870034	3.853469	KEGG	<0.001
GSE18105	p53 signaling pathway	1.029171	0.375248	KEGG	>0.1
GSE21510	p53 signaling pathway	1.144537	0.637388	KEGG	>0.1
GSE28702	p53 signaling pathway	1.662487	2.731669	KEGG	<0.01
GSE29621	p53 signaling pathway	1.598141	2.286094	KEGG	<0.01
GSE29638	p53 signaling pathway	1.598676	2.038665	KEGG	<0.01
GSE39084	p53 signaling pathway	1.474093	1.701995	KEGG	<0.05
GSE71187	p53 signaling pathway	1.287595	1.036156	KEGG	<0.1
GSE72970	p53 signaling pathway	1.889802	4.666152	KEGG	<0.001
GSE87211	p53 signaling pathway	1.853278	3.400955	KEGG	<0.001
TCGA	p53 signaling pathway	-0.9367	0.217669	KEGG	>0.1
GSE103479	Cysteine and methionine metabolism	0.817019	0.122087	KEGG	>0.1
GSE14333	Cysteine and methionine metabolism	1.712179	2.943739	KEGG	<0.01

GSE17536	Cysteine and methionine metabolism	1.288471	1.029384	KEGG	<0.1
GSE18105	Cysteine and methionine metabolism	1.839938	4.1236	KEGG	<0.001
GSE21510	Cysteine and methionine metabolism	1.816171	4.01243	KEGG	<0.001
GSE28702	Cysteine and methionine metabolism	1.615634	1.94917	KEGG	<0.05
GSE29621	Cysteine and methionine metabolism	1.421574	1.5325	KEGG	<0.05
GSE29638	Cysteine and methionine metabolism	2.058697	3.618888	KEGG	<0.001
GSE39084	Cysteine and methionine metabolism	1.889118	3.856805	KEGG	<0.001
GSE71187	Cysteine and methionine metabolism	1.125439	0.60656	KEGG	>0.1
GSE72970	Cysteine and methionine metabolism	1.398621	1.293731	KEGG	<0.1
GSE87211	Cysteine and methionine metabolism	1.449552	1.454303	KEGG	<0.05
TCGA	Cysteine and methionine metabolism	1.716247	2.761752	KEGG	<0.01
GSE103479	Ferroptosis	0.812225	0.124557	KEGG	>0.1
GSE14333	Ferroptosis	1.481052	1.549301	KEGG	<0.05
GSE17536	Ferroptosis	1.942613	3.251025	KEGG	<0.001
GSE18105	Ferroptosis	1.610064	2.236698	KEGG	<0.01
GSE21510	Ferroptosis	1.473411	1.377972	KEGG	<0.05
GSE28702	Ferroptosis	1.593105	1.615034	KEGG	<0.05
GSE29621	Ferroptosis	1.17783	0.608309	KEGG	>0.1
GSE29638	Ferroptosis	1.983406	3.156751	KEGG	<0.001
GSE39084	Ferroptosis	1.065588	0.467848	KEGG	>0.1

GSE71187	Ferroptosis	-1.01673	0.361418	KEGG	>0.1
GSE72970	Ferroptosis	1.444385	1.427173	KEGG	<0.05
GSE87211	Ferroptosis	1.352639	1.022771	KEGG	<0.1
TCGA	Ferroptosis	-0.95093	0.275858	KEGG	>0.1
GSE103479	G2M checkpoint	1.285603	1.24374	HALLMARK	<0.1
GSE14333	G2M checkpoint	2.969426	10	HALLMARK	<0.001
GSE17536	G2M checkpoint	3.243036	10	HALLMARK	<0.001
GSE18105	G2M checkpoint	2.595336	10	HALLMARK	<0.001
GSE21510	G2M checkpoint	2.663865	10	HALLMARK	<0.001
GSE28702	G2M checkpoint	2.643089	10	HALLMARK	<0.001
GSE29621	G2M checkpoint	2.488529	10	HALLMARK	<0.001
GSE29638	G2M checkpoint	3.975135	10	HALLMARK	<0.001
GSE39084	G2M checkpoint	2.988411	10	HALLMARK	<0.001
GSE71187	G2M checkpoint	2.878566	10	HALLMARK	<0.001
GSE72970	G2M checkpoint	2.74451	10	HALLMARK	<0.001
GSE87211	G2M checkpoint	2.833195	10	HALLMARK	<0.001
TCGA	G2M checkpoint	2.674323	10	HALLMARK	<0.001
GSE103479	MYC targets	1.232416	0.982178	HALLMARK	>0.1
GSE14333	MYC targets	3.197187	10	HALLMARK	<0.001
GSE17536	MYC targets	3.148223	10	HALLMARK	<0.001

GSE18105	MYC targets	2.553845	10	HALLMARK	<0.001
GSE21510	MYC targets	2.625904	10	HALLMARK	<0.001
GSE28702	MYC targets	2.987661	10	HALLMARK	<0.001
GSE29621	MYC targets	2.797056	10	HALLMARK	<0.001
GSE29638	MYC targets	4.194264	10	HALLMARK	<0.001
GSE39084	MYC targets	3.350083	10	HALLMARK	<0.001
GSE71187	MYC targets	3.559118	10	HALLMARK	<0.001
GSE72970	MYC targets	3.101578	10	HALLMARK	<0.001
GSE87211	MYC targets	3.698531	10	HALLMARK	<0.001
TCGA	MYC targets	3.055546	10	HALLMARK	<0.001
GSE103479	E2F targets	1.104996	0.56161	HALLMARK	>0.1
GSE14333	E2F targets	3.009145	10	HALLMARK	<0.001
GSE17536	E2F targets	2.914393	10	HALLMARK	<0.001
GSE18105	E2F targets	2.426012	10	HALLMARK	<0.001
GSE21510	E2F targets	2.45617	10	HALLMARK	<0.001
GSE28702	E2F targets	2.925333	10	HALLMARK	<0.001
GSE29621	E2F targets	2.653296	10	HALLMARK	<0.001
GSE29638	E2F targets	4.036317	10	HALLMARK	<0.001
GSE39084	E2F targets	2.973394	10	HALLMARK	<0.001
GSE71187	E2F targets	3.364688	10	HALLMARK	<0.001

GSE72970	E2F targets	2.954132	10	HALLMARK	<0.001
GSE87211	E2F targets	3.349595	10	HALLMARK	<0.001
TCGA	E2F targets	2.827689	10	HALLMARK	<0.001
GSE103479	Vitamin B6 metabolic process	1.609694	2.603681	GO:BP	<0.01
GSE14333	Vitamin B6 metabolic process	1.57192	2.441643	GO:BP	<0.01
GSE17536	Vitamin B6 metabolic process	0.929853	0.200542	GO:BP	>0.1
GSE18105	Vitamin B6 metabolic process	1.389022	1.588733	GO:BP	<0.05
GSE21510	Vitamin B6 metabolic process	1.510154	2.199278	GO:BP	<0.01
GSE28702	Vitamin B6 metabolic process	1.013935	0.358138	GO:BP	>0.1
GSE29621	Vitamin B6 metabolic process	1.376273	1.502333	GO:BP	<0.05
GSE29638	Vitamin B6 metabolic process	1.570241	2.055182	GO:BP	<0.01
GSE39084	Vitamin B6 metabolic process	1.577845	2.740755	GO:BP	<0.01
GSE71187	Vitamin B6 metabolic process	1.122987	0.648456	GO:BP	>0.1
GSE72970	Vitamin B6 metabolic process	1.511371	2.158606	GO:BP	<0.01
GSE87211	Vitamin B6 metabolic process	1.517521	2.300809	GO:BP	<0.01
TCGA	Vitamin B6 metabolic process	0.836694	0.073069	GO:BP	>0.1
GSE103479	Cell redox homeostasis	1.487066	1.374519	GO:BP	<0.05
GSE14333	Cell redox homeostasis	2.052467	4.788692	GO:BP	<0.001
GSE17536	Cell redox homeostasis	1.810364	2.681567	GO:BP	<0.01
GSE18105	Cell redox homeostasis	1.747235	2.847591	GO:BP	<0.01

GSE21510	Cell redox homeostasis	1.638203	2.37767	GO:BP	<0.01
GSE28702	Cell redox homeostasis	1.845212	2.952641	GO:BP	<0.01
GSE29621	Cell redox homeostasis	1.628431	2.247824	GO:BP	<0.01
GSE29638	Cell redox homeostasis	1.252078	0.807462	GO:BP	>0.1
GSE39084	Cell redox homeostasis	1.731122	2.732948	GO:BP	<0.01
GSE71187	Cell redox homeostasis	1.327684	1.040157	GO:BP	<0.1
GSE72970	Cell redox homeostasis	1.686621	2.411709	GO:BP	<0.01
GSE87211	Cell redox homeostasis	0.820652	0.117522	GO:BP	>0.1
TCGA	Cell redox homeostasis	1.0144	0.412349	GO:BP	>0.1
GSE103479	Cellular response to oxidative stress	1.236778	1.084255	GO:BP	<0.1
GSE14333	Cellular response to oxidative stress	1.432907	2.774764	GO:BP	<0.01
GSE17536	Cellular response to oxidative stress	1.325643	1.731437	GO:BP	<0.05
GSE18105	Cellular response to oxidative stress	1.29117	1.541964	GO:BP	<0.05
GSE21510	Cellular response to oxidative stress	1.248745	1.184006	GO:BP	<0.1
GSE28702	Cellular response to oxidative stress	1.571383	3.475451	GO:BP	<0.001
GSE29621	Cellular response to oxidative stress	1.417776	2.498846	GO:BP	<0.01
GSE29638	Cellular response to oxidative stress	1.430855	1.93028	GO:BP	<0.05
GSE39084	Cellular response to oxidative stress	-1.24932	1.276275	GO:BP	<0.1
GSE71187	Cellular response to oxidative stress	-1.4279	2.256173	GO:BP	<0.01
GSE72970	Cellular response to oxidative stress	1.365734	1.724551	GO:BP	<0.05

GSE87211	Cellular response to oxidative stress	1.406447	2.229259	GO:BP	<0.01
TCGA	Cellular response to oxidative stress	-1.3596	1.961127	GO:BP	<0.05

Table S9. The correlation between the IC50 value of drugs related to ferroptosis and the expression level of NSUN2

Dataset	Drug	GSE103479	GSE14333	GSE17536	GSE18105	GSE21510	GSE28702	GSE29621	GSE29638	GSE39084	GSE71187	GSE72970	GSE87211	TCGA
GDSC2	Oxaliplatin_1089	-0.060426647	0.421488035	0.198037461	0.381008757	0.359851793	0.230166115	0.356099801	0.05089197	0.292859568	0.261395073	0.190958391	0.248845638	0.210115929
	Oxaliplatin_1806	-0.156953716	0.328629783	0.124544251	0.106103339	0.103848417	0.114241806	0.299669543	-0.046302837	0.124527081	0.169105359	0.098343595	0.093671689	0.134889457
CTRP2	Erastin	0.099282391	0.280068672	0.335351686	0.260819202	0.249245149	0.311918688	0.473807825	0.21250588	0.315611258	0.240188287	0.229804916	0.158184594	-0.081596133
	ML162	0.093823862	0.5127313	0.401963938	0.317616698	0.260324007	0.40951391	0.522296239	0.686379891	0.521416256	0.232386689	0.454019413	0.57167422	0.209995079

Table S10. The significance of the correlation between the IC50 value of drugs related to ferroptosis and the expression level of NSUN2

Data set	Drug	GSE103479	GSE14333	GSE17536	GSE18105	GSE21510	GSE28702	GSE29621	GSE29638	GSE39084	GSE71187	GSE72970	GSE87211	TCGA
GDSC2	Oxaliplatin_1089	0.453650034	3.80E-11	0.008235203	3.70E-05	7.05E-06	0.036321885	0.003598707	0.725598669	0.01388117	0.000280013	0.033630233	1.58E-06	1.73E-06

	Oxaliplatin _1806	0.05037 9205	4.32E-07	0.09860 5779	0.26771 2531	0.20909 5103	0.30377 1307	0.01530 1948	0.74949 8528	0.30436 7091	0.02000 9481	0.27718 9948	0.07467 5726	0.00229 0609
CTR P2	Erastin	0.21753 3706	1.93E-05	5.05E-06	0.01111 6641	0.00543 4984	0.00409 686	6.70E-05	0.13844 1763	0.00778 0651	0.01663 5844	0.01024 2255	0.00250 7141	0.08346 6771
	ML162	0.24401 6241	1.49E-16	2.93E-08	0.00181 2203	0.00363 7873	0.00012 0899	8.10E-06	3.75E-08	3.69E-06	0.02063 2843	1.18E-07	6.95E-33	6.88E-06

Table S11. The keg genes of the ferroptosis pathway

Ferroptosis activators	Ferroptosis suppressors
TFRC	SLC7A11
STEAP3	GPX4
DMT1	FTH1
ACO1	FTL
IREB2	NFE2L2
ACSL4	HMOX1
LPCAT3	SLC40A1
ALOX15	FSP1
POR	GCH1
SAT1	DHODH
CHAC1	
ATP13A2	

KEAP1	
BACH1	
TP53	
ATF4	
HIF1A	
NOX1	
NOX4	

Table S12. The enrichment of the ferroptosis pathway in different NSUN2 expression pattern cell clusters of single-cell data

Dataset	NSUN2-cell	NSUN2+cell	pathway
GSE232525_Naive	0.892032383	-1.199223261	ferroptosis_activators
GSE232525_Treat	0.999768099	-1.477403519	ferroptosis_activators
GSE299427_Naive	1.718741899	1.50021898	ferroptosis_activators
GSE299427_Treat	2	0.39412654	ferroptosis_activators
GSE232525_Naive	-0.018144304	1.091077462	ferroptosis_suppressors
GSE232525_Treat	-2	1.499726644	ferroptosis_suppressors
GSE299427_Naive	0.924767933	1.615246537	ferroptosis_suppressors
GSE299427_Treat	0.240342657	1.696774562	ferroptosis_suppressors

Table S13. The correlation between the induction of ferroptosis-related drug sensitivity and the expression level of NSUN2 in spatial transcriptomics

Gene	Signature	Rho	P_value	n	sample
NSUN2	Oxaliplatin2	-0.1083	5.14E-05	1392	C1
NSUN2	ML162	-0.21514	1.66E-16	1392	C1
NSUN2	Erastin	0.000576	0.982869	1392	C1
NSUN2	Oxaliplatin2	-0.17636	3.08E-14	1500	C2
NSUN2	Oxaliplatin1	0.185444	4.52E-13	1500	C2
NSUN2	Oxaliplatin1	0.12148	2.87E-09	2375	C3
NSUN2	ML162	-0.22564	9.07E-19	1500	C2
NSUN2	Erastin	-0.26671	9.75E-24	1500	C2
NSUN2	Oxaliplatin2	-0.28638	4.56E-46	2375	C3
NSUN2	ML162	-0.29743	1.02E-49	2375	C3
NSUN2	Oxaliplatin1	0.079005	0.051136	610	C4
NSUN2	Erastin	-0.19648	4.26E-22	2375	C3
NSUN2	Oxaliplatin2	-0.01768	0.663008	610	C4
NSUN2	Oxaliplatin1	-0.00419	0.875795	1392	C1
NSUN2	ML162	-0.14997	9.02E-12	610	C4
NSUN2	Erastin	0.053283	0.188768	610	C4

Table S14. The correlation between the ferroptosis suppressors in different bulk cohorts and the expression level of NSUN2

Gene	GSE103479	GSE14333	GSE17536	GSE18105	GSE21510	GSE25071	GSE28702	GSE29621	GSE29638	GSE39084	GSE71187	GSE72970	GSE87211	TCGA	median
DHODH	-0.216972085	0.353322474	0.318889513	0.353897789	0.403822836	0.390506157	0.338788797	0.172068984	0.510006629	0.332893246	0.433673856	0.374630453	0.365503457	0.374378047	0.359701
SLC7A11	-0.006914786	0.33937987	0.248531959	0.50028177	0.529370213	-0.075782502	0.17663085	0.453532794	0.502606806	0.280738765	0.566732994	0.278139173	0.461345173	0.322933502	0.331157
GCH1	0.15403582	0.474767847	0.399772845	0.196083742	0.098239864	-0.092836462	0.402246236	0.526803205	-0.121586789	0.178155036	0.220616744	0.218330556	0.084800839	-0.018995669	0.187119
NFE2L2	0.119155288	0.200375941	0.236482657	0.246094644	0.08986079	0.031416634	0.043104098	0.060114502	0.172419483	0.017597759	0.149481132	0.338182692	-0.068850132	-0.06329584	0.104508
GPX4	0.236596564	0.089807758	0.061777895	0.112011173	0.152440415	0.15261906	-0.143624513	-0.035582042	-0.142149972	-0.113865646	0.101397806	-0.074104095	-0.018375357	-0.01529906	0.023239
FTL	0.179036361	-0.178696133	-0.043890832	0.269447096	0.195579645	0.129110661	-0.161826979	-0.073958358	-0.357898075	-0.309039557	0.070627817	0.011052934	-0.045373792	0.085955071	-0.01642
FTH1	-0.062191383	0.108839953	0.045023547	-0.140975883	-0.18905765	0.195662774	0.078604242	0.071340828	-0.151525439	-0.172869195	0.021806258	0.070626748	-0.312255397	-0.134122047	-0.02019
HMOX1	-0.34933945	-0.229798328	0.195560836	-0.429831193	-0.512960877	0.014180071	0.189502554	0.011105122	-0.403652849	0.072068556	-0.108230879	0.005066139	-0.03742938	-0.258251046	-0.07283

SLC40A1	0.207545061	-0.18320673	-0.119729379	0.009059264	-0.176179515	-0.172823086	0.048470421	-0.278660928	-0.004958862	-0.241100858	-0.229891395	-0.113920888	-0.305399496	-0.16569579	-0.16926

Table S15. The significance of the correlation between the ferroptosis suppressors in different bulk cohorts and the expression level of NSUN2

Gene	GSE103479	GSE14333	GSE17536	GSE18105	GSE21510	GSE25071	GSE28702	GSE29621	GSE29638	GSE39084	GSE71187	GSE72970	GSE87211	TCGA
DHODH	0.006514893	4.78E-08	1.52E-05	0.000138733	3.59E-07	0.005051638	0.00173085	0.170501486	0.000154717	0.004865174	4.56E-10	1.81E-05	6.48E-13	2.22E-18
SLC7A11	0.931727098	1.70E-07	0.000851253	2.26E-08	4.60E-12	0.600930494	0.110184132	0.000148162	0.000199583	0.018568009	1.89E-17	0.001761088	1.56E-20	8.09E-14
GCH1	0.054869505	4.17E-14	3.54E-08	0.03915516	0.234879985	0.521369081	0.000163508	6.55E-06	0.400275119	0.140065795	0.002284343	0.014847875	0.106742954	0.668981145
NFE2L2	0.138456515	0.002475176	0.001529761	0.009225357	0.277419934	0.828532747	0.698814376	0.634298633	0.231172057	0.885031798	0.040078238	0.000122273	0.190601957	0.153886108
GPX4	0.00294269	0.178515376	0.414009176	0.241824149	0.064368542	0.290010706	0.195194314	0.778407865	0.324751217	0.347950451	0.165038855	0.413382051	0.727149712	0.730595065
FTL	0.025333455	0.007076821	0.561866121	0.004239669	0.017209168	0.371532038	0.143848688	0.558208531	0.010715012	0.009238199	0.33417571	0.903027747	0.388715452	0.052616503
FTH1	0.440546949	0.10267905	0.551805253	0.139986581	0.021375921	0.17327523	0.479969063	0.572258536	0.293521886	0.152406028	0.765830527	0.435699878	1.19E-09	0.002427761
HMOX1	7.83E-06	0.000497335	0.009092135	2.50E-06	2.63E-11	0.922141117	0.086189046	0.930037378	0.003650707	0.553258994	0.138229697	0.955466767	0.47713688	3.37E-09

SLC40A1	0.009328888	0.00573873	0.112439037	0.924817826	0.03219938	0.230065223	0.663456804	0.024590848	0.972735407	0.044363461	0.00146136	0.207731626	2.83E-09	0.000173297

Table S16. The Cox regression results of the DHODH gene on prognosis in different clinical cohorts

Dataset	Gene	HR	CI_lower	CI_upper	PValue	state
GSE29621	DHODH	2.039151	0.804348	5.169575	0.133293	OS
GSE72970	DHODH	1.294886	0.743928	2.253887	0.360783	OS
GSE87211	DHODH	1.717853	1.029715	3.012121	0.048967	OS
GSE14333	DHODH	1.56389	1.112955	2.19753	0.009978	DFS
GSE72970	DHODH	1.599939	0.991587	2.581523	0.054185	DFS
GSE87211	DHODH	1.522735	0.975901	2.375981	0.063954	DFS
GSE103479	DHODH	1.475182	0.633809	3.433465	0.367058	DFS

Table S17. The correlation between novel ferroptosis suppressors in different bulk cohorts and the expression level of NSUN2

	GSE103479	GSE14333	GSE17536	GSE18105	GSE21510	GSE25071	GSE28702	GSE29621	GSE29638	GSE39084	GSE71187	GSE72970	GSE87211	TCGA
GCH1	0.154036	0.474768	0.399773	0.196084	0.09824	-0.09284	0.402246	0.526803	-0.12159	0.178155	0.220617	0.218331	0.084801	-0.019
AIFM2	0.396818	0.377908	0.08731	0.23889	0.12702	-0.06796	0.131882	0.177296	0.009192	0.088705	0.20853	0.219708	0.144428	0.006542

GSTP1	0.002354	0.177728	0.321981	0.099248	0.143149	-0.03472	-0.0374	0.107465	0.095072	0.138454	0.168225	0.172632	0.019438	-0.00969

Table S18. The significance of the correlation between novel ferroptosis suppressors in different bulk cohorts and the expression level of NSUN2

	GSE103479	GSE14333	GSE17536	GSE18105	GSE21510	GSE25071	GSE28702	GSE29621	GSE29638	GSE39084	GSE71187	GSE72970	GSE87211	TCGA
GCH1	0.05487	4.17E-14	3.54E-08	0.039155	0.23488	0.521369	0.000164	6.55E-06	0.400275	0.140066	0.002284	0.014848	0.106743	0.668981
AIFM2	2.92E-07	4.39E-09	0.247861	0.020405	0.161508	0.653592	0.234644	0.157687	0.950559	0.465243	0.038328	0.014214	0.005839	0.889802
GSTP1	0.976735	0.007398	1.24E-05	0.34124	0.114212	0.818811	0.737094	0.394174	0.520371	0.25302	0.096024	0.0552	0.71205	0.837321
